# Target engagement and immunogenicity of an active immunotherapeutic targeting pathological α-synuclein: a phase 1 placebo-controlled trial

**DOI:** 10.1038/s41591-024-03101-8

**Published:** 2024-06-20

**Authors:** Pepijn Eijsvogel, Pinaki Misra, Luis Concha-Marambio, Justin D. Boyd, Shuang Ding, Lauren Fedor, Yueh-Ting Hsieh, Yu Shuang Sun, Madeline M. Vroom, Carly M. Farris, Yihua Ma, Marieke L. de Kam, Igor Radanovic, Maurits F. J. M. Vissers, Dario Mirski, Ghazal Shareghi, Mohammad Shahnawaz, Wolfgang Singer, Philip Kremer, Geert Jan Groeneveld, Hui Jing Yu, Jean-Cosme Dodart

**Affiliations:** 1 Centre for Human Drug Research and Leiden University Medical Centre, Leiden, The Netherlands; 2 Department of Neurology, Mayo Clinic, Rochester, MN USA; 3 R&D Unit, Amprion Inc, San Diego, CA USA; 4 Vaxxinity Inc, Merritt Island, FL USA; 5 Centre for Human Drug Research Leiden, Leiden, The Netherlands; 6 Mitchell Center for Alzheimer’s Disease and Related Brain Disorders, University of Texas McGovern Medical School, Houston, TX USA

**Keywords:** Parkinson's disease, Drug development

## Abstract

Investigational therapeutics that target toxic species of α-synuclein (αSyn) aim to slow down or halt disease progression in patients with Parkinson’s disease (PD). Here this 44-week, randomized, placebo-controlled, double-blind, single-center phase 1 study investigated safety, tolerability and immunogenicity of UB-312, an active immunotherapeutic targeting pathological αSyn, in patients with PD. The primary outcome measures were adverse event frequency and change in anti-αSyn antibody titers in blood and cerebrospinal fluid (CSF). Exploratory outcomes were changes in clinical scales and biomarker-based target engagement as measured by seed amplification assays. Twenty patients were randomized 7:3 (UB-312:placebo) into 300/100/100 μg or 300/300/300 μg (weeks 1, 5 and 13) intramuscular prime-boost dose groups. Safety was similar across groups; adverse events were mostly mild and transient. Two patients experienced three serious adverse events in total, one possibly treatment related; all resolved without sequalae. Anti-αSyn antibodies in serum from 12/13 and CSF from 5/13 patients who received three UB-312 doses confirmed immunogenicity. Mean serum titers (in log-dilution factor) increased from baseline by 1.398 and 1.354, and peaked at week 29 at 2.520 and 2.133, for 300/100/100 μg and 300/300/300 μg, respectively. CSF titers were 0 at baseline and were 0.182 and 0.032 at week 21, respectively. Exploratory analyses showed no statistical differences in clinical scales but a significant reduction of αSyn seeds in CSF of a subset of UB-312-treated patients. These data support further UB-312 development. ClinicalTrials.gov:NCT04075318.

## Abstract (web summary)

A first-in-patient trial of UB-312, an active immunotherapeutic for Parkinson’s disease, was well tolerated and elicited antibodies that engage misfolded α-synuclein.

## Main

Parkinson’s disease (PD) is characterized by progressive deterioration of motor, cognitive, behavioral and autonomic function^1^. Mechanisms of dopaminergic cell loss in PD are not fully understood; however, α-synuclein (αSyn) has a central role in neurodegeneration. Expressed primarily in presynaptic terminals, αSyn is involved in synaptic vesicle trafficking and modulation and neurotransmitter regulation^2,3^. Duplications, point mutations or single nucleotide polymorphisms in *SNCA*, encoding αSyn, contribute to PD susceptibility; the mutated forms of the protein have altered structural configurations that promote pathological aggregation^4,5^. While such mutations are rare, αSyn aggregates in the form of Lewy bodies (LB) are common neuropathological hallmarks of PD. Moreover, preformed fibrils of αSyn can induce formation of LB-like inclusions and cellular dysfunction in cell-based assays and preclinical animal models^3,6^. Together, these data strongly suggest that targeting pathological, aggregation-prone forms of αSyn has therapeutic potential.

There are no approved disease-modifying therapies for PD. Passive and active immunotherapies targeting αSyn (that is, delivery of monoclonal antibodies or vaccination to raise an endogenous immune response, respectively) can ameliorate αSyn pathology and functional deficits in mouse models of PD, and both approaches are now in clinical development^7–9^. These approaches have exhibited promising results in phase 1 clinical trials^7,8,10,11^. Two phase 2 clinical trials recently failed to demonstrate efficacy of monoclonal antibodies against αSyn^12,13^, but as was experienced in Alzheimer’s disease, early failure to demonstrate clinical efficacy does not necessarily invalidate the therapeutic target or investigational drug^14^. Trial design is crucial and elements such as appropriate patient selection, choice of clinical scales and inclusion of relevant biomarkers require careful refinement. The importance of biomarkers to test target engagement in Alzheimer’s disease drug development was illustrated with validation of amyloid positron emission tomography imaging to monitor effects on brain pathology^15^, which accelerated decision making in many anti-amyloid drug development programs. However, convincing biomarkers of target engagement to support clinical trials in patients with PD have so far been lacking.

Successful vaccination against endogenous targets requires overcoming immune tolerance to generate a humoral antibody response while avoiding T cell-mediated cytotoxicity; both are dependent on how the relevant epitope is presented to the immune system. A novel vaccine carrier platform utilizing proprietary synthetic T-helper peptides linked to target epitopes demonstrated potential to achieve these aims, inducing a targeted B cell humoral response without T cell-mediated toxicity^16^. UB-312 was selected from over 60 synthetic peptide immunogens, and has demonstrated high immunogenicity in preclinical studies across species (unpublished data). Notably, antibodies induced by UB-312 selectively targeted pathological oligomeric and fibrillar αSyn forms, binding specifically to αSyn inclusions in postmortem brain sections from patients with PD, dementia with LBs and multiple system atrophy (MSA)^17^. Furthermore, UB-312-derived antibodies exhibited neuroprotective effects in vitro, reduced αSyn in the brain and the gut and prevented motor function deficits in a transgenic synucleinopathy mouse model^18^.

In Part A of this phase 1 study, escalating doses of UB-312 were tested versus placebo in healthy volunteers aged 40–85 years, as previously reported^19^. UB-312 was considered safe and well tolerated up to 300/300/300 μg three-dose ‘prime-boost’ regimen, with most adverse events being mild and transient. UB-312 triggered dose- and time-dependent antibody production, with anti-αSyn antibodies detectable in both serum and cerebrospinal fluid (CSF) for all participants receiving the 300/300/300 μg prime-boost UB-312 regimen, and an average CSF/serum ratio of 0.2%. Serum and CSF αSyn concentrations were not altered by treatment, consistent with preferential binding of UB-312-derived antibodies to pathological forms of αSyn and poor binding to monomeric αSyn^17^. The 300/100/100 μg and 300/300/300 μg prime-boost dose regimens were selected based on safety and immunogenicity profile for further evaluation in patients with PD.

This phase 1 study Part B was designed to assess the safety, tolerability and immunogenicity of the two chosen UB-312 regimens in patients with PD. To address the unmet need of a biomarker for target engagement by immunotherapies in clinical trials in patients with PD, we also investigated use of the αSyn seed amplification assay (αSyn-SAA), which is able to detect small amounts of pathological αSyn and is increasingly used to support diagnosis of PD^20^. Application of αSyn-SAA to the assessment of target engagement by UB-312-induced antibodies was an exploratory biomarker endpoint. Other exploratory outcome measurements included cognitive and PD-related clinical efficacy assessments.

## Results

### Patient disposition

**Fig. 1:**
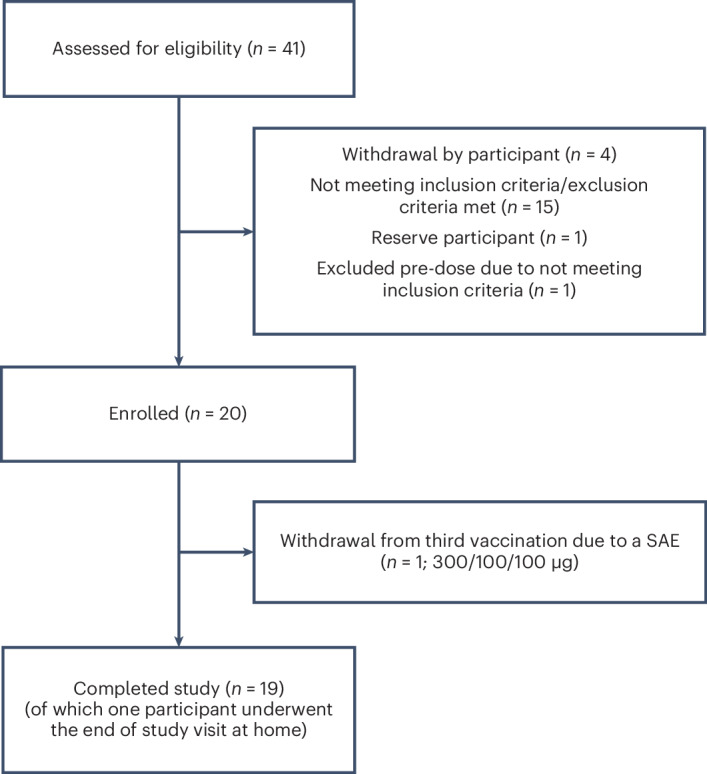
Patient disposition. Enrolled patients were randomized to placebo (*n* = 6), UB-312 300/100/100 μg (*n* = 7) and UB-312 300/300/300 μg (*n* = 7) treatment groups. The PP population comprised 20 participants up to week 13 and 19 thereafter.

Recruitment occurred from 27 October 2021 to 6 April 2022. Among 41 participants screened, 15 were ineligible based on inclusion or exclusion criteria, and four withdrew after screening (Fig. 1). Between 11 January 2022 and 27 April 2022, 21 participants were randomized to either UB-312 or placebo, and one was planned as reserve participant. One participant was excluded before the first vaccination due to elevated C-reactive protein level. Twenty participants received the first and second injection, and 19 received the third; one participant in the 300/100/100 μg cohort did not due to a serious adverse event (SAE). All participants completed the follow-up visits. The modified intention-to-treat (mITT) population consisted of all 20 participants. The per-protocol (PP) population comprised 20 participants up to week 13; after one participant did not receive the third vaccination, the PP population was 19 of 20 participants. Baseline characteristics were comparable between groups, including Hoehn and Yahr (H&Y) stage and PD duration in years (Table 1).

**Table 1:** Demographic baseline data in all participants and per treatment group

N	All participants	UB-312	UB-312	Placebo
		300/100/100 μg	300/300/300 μg	
	20	7	7	6
Age, years				
Mean (s.d.)	64.1 (9.9)	67.4 (12.3)	63.4 (9.0)	61.0 (8.0)
Min; Max	44; 83	44; 83	50; 75	51; 73
Height, cm				
Mean (s.d.)	177.8 (7.9)	177.4 (4.8)	176.1 (10.3)	180.1 (8.4)
Min; Max	159.8; 192.2	169.4; 182.0	159.8; 188.7	166.1; 192.2
Weight, kg				
Mean (s.d.)	79.7 (10.5)	81.5 (10.3)	75.6 (11.1)	82.3 (10.3)
Min; Max	62.0; 101.6	69.3; 101.6	62.0; 96.3	66.2; 90.9
BMI, kg m^−2^				
Mean (s.d.)	25.2 (2.9)	25.9 (2.6)	24.4 (3.1)	25.4 (3.1)
Min; Max	19.7; 30.7	22.8; 30.7	20.8; 29.4	19.7; 28.1
Sex, *n* (%)				
Female	4 (20.0)	1 (14.3)	2 (28.6)	1 (16.7)
Male	16 (80.0)	6 (85.7)	5 (71.4)	5 (83.3)
Race, *n* (%)				
White	20 (100)	7 (100)	7 (100%)	6 (100)
H&Y^a^, *n* (%)				
0 (Asymptomatic)	0 (0)	0 (0)	0 (0)	0 (0)
1 (Unilateral involvement only)	2 (10.0)	0 (0)	2 (28.6)	0 (0)
2 (Bilateral involvement without balance impairment)	16 (80.0)	7 (100)	4 (57.1)	5 (83.3)
3 (Mild-to-moderate involvement)	2 (10.0)	0 (0)	1 (14.3)	1 (16.7)
MDS–UPDRS part II total score^a^				
Mean (s.d.)	10.5 (6.23)	14.1 (6.77)	8.4 (5.38)	8.5 (5.32)
Min; Max	1; 27	6; 27	1; 15	1; 16
MDS–UPDRS part III total score^a^				
Mean (s.d.)	33.5 (15.37)	37.1 (14.72)	37.4 (19.92)	24.7 (5.28)
Min; Max	8; 60	23; 62	8; 60	18; 31
MoCA total score^a^				
Mean (s.d.)	27.1 (2.17)	26.9 (2.12)	27.0 (2.71)	27.5 (1.87)
Min; Max	21; 30	23;29	21; 29	25; 30
Parkinson duration, years				
Mean (s.d.)	6.8 (4.3)	7.4 (3.8)	7.6 (5.4)	5.0 (3.5)
Min; Max	1; 16	2;13	2; 16	1; 11
Anti-Parkinson drugs, *n* (%)				
Levodopa	18 (90.0)	7 (100)	5 (71.4)	6 (100)
Dopamine agonists	10 (50.0)	5 (71.4)	3 (42.9)	2 (33.3)
MAO-B inhibitors	0 (0)	0 (0)	0 (0)	0 (0)

Baseline characteristics were comparable between study groups, including H&Y stage and duration of PD.

^a^Measured prevaccination on day 1.

### Primary outcomes: safety, tolerability and immunogenicity

Headache, local pain after lumbar puncture and fatigue were the most frequently reported treatment-emergent adverse events (TEAEs) (Table 2). TEAEs appeared to occur equally after administration of UB-312 (14 of 14 participants) and placebo (5 of 6 participants) (Table 2). Most TEAEs were considered either mild or moderate after administration of UB-312, comparable to placebo. Three SAEs were reported, of which one—a deep venous thrombosis of the left leg 50 days after the second administration of UB-312 300/100/100 μg—was considered possibly related, due to the timing of onset. There were no safety signals in assessments including electrocardiogram (ECG), vital signs and blood and urine assessments. There was no difference in either physician- or participant-reported tolerability within 7 days after each administration of UB-312 compared with placebo. No postvaccination brain magnetic resonance imagings (MRIs) were performed.

**Table 2:** Summary of all TEAEs per treatment group and pooled UB-312 cohorts

Treatment group	UB-312							
	300/100/100 μg		300/300/300 μg		Pooled		Placebo	
Number of participants	7		7		14		6	
System organ class/preferred term	Events	Participants	Events	Participants	Events	Participants	Events	Participants
	*N*	*N* (%)	*N*	*N* (%)	*N*	*N* (%)	*N*	*N* (%)
Any events	59	7 (100.0)	42	7 (100.0)	101	14 (100.0)	20	5 (83.3)
Cardiac disorders:	1	1 (14.3)	2	1 (14.3)	3	2 (14.3)	2	1 (16.7)
Arrhythmia	–	–	–	–	–	–	1	1 (16.7)
Atrial fibrillation	–	–	–	–	–	–	1	1 (16.7)
Palpitations	1	1 (14.3)	2	1 (14.3)	3	2 (14.3)	–	–
Eye disorders:	1	1 (14.3)	–	–	1	1 (7.1)	–	–
Vision blurred	1	1 (14.3)	–	–	1	1 (7.1)	–	–
Gastrointestinal disorders:	2	2 (28.6)	2	1 (14·3)	4	3 (21·4)	1	1 (16.7)
Diarrhea	2	2 (28.6)	1	1 (14.3)	3	3 (21.4)	–	–
Nausea	–	–	1	1 (14.3)	1	1 (7.1)	1	1 (16.7)
General disorders and administration site conditions:	8	4 (57.1)	4	2 (28.6)	12	6 (42.9)	5	3 (50.0)
Chest pain	1	1 (14.3)	–	–	1	1 (7.1)	–	–
Fatigue	4	3 (42.9)	1	1 (14.3)	5	4 (28.6)	2	1 (16.7)
Injection-site pain	3	2 (28.6)	1	1 (14.3)	4	3 (21.4)	3	2 (33.3)
Malaise	–	–	2	1 (14.3)	2	1 (7.1)	–	–
Infections and infestations:	9	4 (57.1)	10	5 (71.4)	19	9 (64.3)	1	1 (16.7)
Acute sinusitis	–	–	1	1 (14.3)	1	1 (7.1)	–	–
Coronavirus disease 2019	2	2 (28.6)	2	2 (28.6)	4	4 (28.6)	–	–
Gastroenteritis	–	–	1	1 (14.3)	1	1 (7.1)	–	–
Pharyngitis	–	–	1	1 (14.3)	1	1 (7.1)	–	–
Pneumonia	1	1 (14.3)	–	–	1	1 (7.1)	–	–
Post-acute coronavirus disease 2019 syndrome	1	1 (14.3)	1	1 (14.3)	2	2 (14.3)		
Respiratory tract infection	–	–	1	1 (14.3)	1	1 (7.1)	–	–
Rhinitis	–	–	1	1 (14.3)	1	1 (7.1)	–	–
Upper respiratory tract infection	2	2 (28.6)	1	1 (14.3)	3	3 (21.4)	1	1 (16.7)
Urinary tract infection	1	1 (14.3)	1	1 (14.3)	2	2 (14.3)	–	–
Urosepsis	1	1 (14.3)	–	–	1	1 (7.1)	–	–
Viral infection	1	1 (14.3)	–	–	1	1 (7.1)	–	–
Injury, poisoning and procedural complications:	8	6 (85.7)	1	1 (14.3)	9	7 (50)	3	2 (33.3)
Clavicle fracture	1	1 (14.3)	–	–	1	1 (7.1)	–	–
Concussion	1	1 (14.3)	–	–	1	1 (7.1)	1	1 (16.7)
Muscle strain	–	–	–	–	–	–	1	1 (16.7)
Post-lumbar puncture syndrome	1	1 (14.3)	–	–	1	1 (7.1)	–	–
Local pain after lumbar puncture	4	4 (57.1)	1	1 (14.3)	5	5 (35·7)	1	1 (16.7)
Skin laceration	1	1 (14.3)	–	–	1	1 (7.1)	–	–
Investigations:	1	1 (14.3)	–	–	1	1 (7.1)	–	–
Blood pressure systolic increased	1	1 (14.3)	–	–	1	1 (7.1)	–	–
Metabolism and nutrition disorders:	–	–	1	1 (14.3)	1	1 (7.1)	–	–
Hypercholesterolemia	–	–	1	1 (14.3)	1	1 (7.1)	–	–
Musculoskeletal and connective tissue disorders:	8	2 (28.6)	2	2 (28.6)	10	4 (28.6)	–	–
Myalgia	7	2 (28.6)	2	2 (28.6)	9	4 (28.6)	–	–
Plantar fasciitis	1	1 (14.3)	–	–	1	1 (7.1)	–	–
Nervous system disorders:	14	6 (85.7)	13	7 (100.0)	27	13 (92.9)	4	4 (66.7)
Carpal tunnel syndrome	–	–	–	–			1	1 (16.7)
Dizziness	2	2 (28.6)	1	1 (14.3)	3	3 (21.4)	–	–
Headache	10	5 (71.4)	10	6 (85.7)	20	11 (78.6)	3	3 (50.0)
Presyncope	1	1 (14.3)	1	1 (14.3)	2	2 (14.3)	–	–
Sedation	–	–	1	1 (14.3)	1	1 (7.1)	–	–
Somnolence	1	1 (14.3)	–	–	1	1 (7.1)	–	–
Psychiatric disorders:	1	1 (14.3)	2	2 (28.6)	3	3 (21.4)	–	–
Insomnia	1	1 (14.3)	1	1 (14.3)	2	2 (14.3)	–	–
Stress	–	–	1	1 (14.·3)	1	1 (7.1)	–	–
Renal and urinary disorders:	–	–	–	–	–	–	1	1 (16.7)
Renal cyst	–	–	–	–	–	–	1	1 (16.7)
Respiratory thoracic and mediastinal disorders:	1	1 (14.3)	2	2 (28.6)	3	3 (21.4)	1	1 (16.7)
Cough	–	–	1	1 (14.3)	1	1 (7.1)	–	–
Dyspnea	1	1 (14.3)	–	–	1	1 (7.1)	–	–
Epistaxis	–	–	–	–			1	1 (16.7)
Sneezing	–	–	1	1 (14.3)	1	1 (7.1)	–	–
Skin and subcutaneous tissue disorders:	2	1 (14.3)	–	–	2	1 (7.1)	1	1 (16.7)
Erythema	1	1 (14.3)	–	–	1	1 (7.1)	–	–
Photosensitivity reaction	–	–	–	–	–	–	1	1 (16.7)
Rash	1	1 (14.3)	–	–	1	1 (7.1)	–	–
Vascular disorders:	3	3 (42.9)	3	3 (42.9)	6	6 (42.9)	1	1 (16.7)
Deep vein thrombosis	1	1 (14.3)	–	–	1	1 (7.1)	–	–
Orthostatic hypotension	–	–	3	3 (42.9)	3	3 (21.4)	1	1 (16.7)
Peripheral venous disease	2	2 (28.6)	–	–	2	2 (14.3)	–	–

Specific TEAEs are listed by total number of events (*N*), as well as number and percentage (*N* (%)) of participants reporting the specific adverse event. Headache, local pain after lumbar puncture and fatigue were the most frequently reported TEAEs after vaccination with UB-312. TEAEs appeared to occur equally after administration of UB-312 (14 of 14 participants) and placebo (5 of 6 participants).

**Fig. 2:**
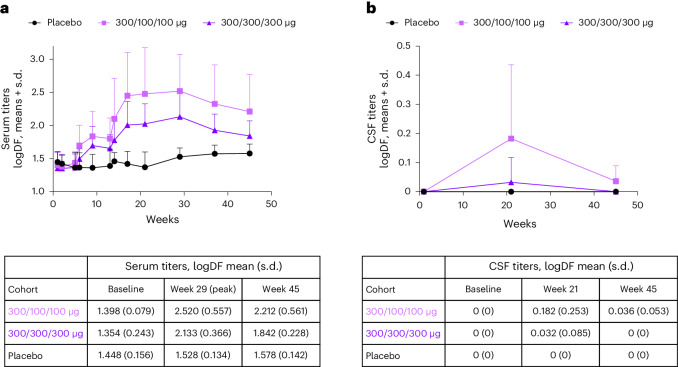
Epitope-specific anti-αSyn antibody titers in blood and CSF. UB-312 or placebo were administered at weeks 1, 5 and 13. **a**, Serum antibody titers increased predominantly after the third vaccination. The definition of seroconversion was met in 12/13 participants receiving all three doses (5/6 in the 300/100/100 μg group and 7/7 in the 300/300/300 μg group). Increases were more pronounced in the 300/100/100 μg treatment group compared with the 300/300/300 μg treatment group. Samples were analyzed in duplicate and data are expressed as logDF mean + s.d. **b**, CSF antibody titers were more pronounced in the 300/100/100 μg treatment group compared with the 300/300/300 μg cohort; levels were detectable in 4/6 and 1/7 participants, respectively. Samples were analyzed in duplicate and data are expressed as logDF mean + s.d. Numbers per group: placebo, *n* = 6; 300/100/100 μg, *n* = 7 up to week 13 and thereafter *n* = 6 until week 45; 300/300/300 μg, *n* = 7. logDF, log-dilution factor.

UB-312 vaccination generated robust and time-dependent serum antibodies against the C-terminal epitope αSyn97–135, with titers peaking at week 29 and remaining greater than baseline values at week 45 (Fig. 2a).

The definition of seroconversion was met for five of six participants after administration of 300/100/100 μg, and all seven participants after administration of 300/300/300 μg who received all three doses of UB-312, for an overall seroconversion rate of 12/13.

Epitope-specific anti-αSyn antibodies were also detectable in CSF, in a total of 5/13 participants who received all three doses; 4/6 in the 300/100/100 µg group and 1/7 in the 300/300/300 μg group. After peaking at week 21 (Fig. 2b), CSF titers at week 45 were measurable in only two participants. UB-312 vaccination did not lead to the generation of antibodies against full-length (FL) αSyn compared with placebo.

### Exploratory outcomes

Total scores of the Montreal Cognitive Assessment (MoCA) and the Movement Disorder Society–Unified Parkinson’s Disease Rating Scale (MDS–UPDRS) part II (MDS–UPDRS-II) and part III (MDS–UPDRS-III) were generally stable during the study, with no statistical differences between groups (Supplementary Figs. 1 and 2).

**Extended Data Fig. 1:**
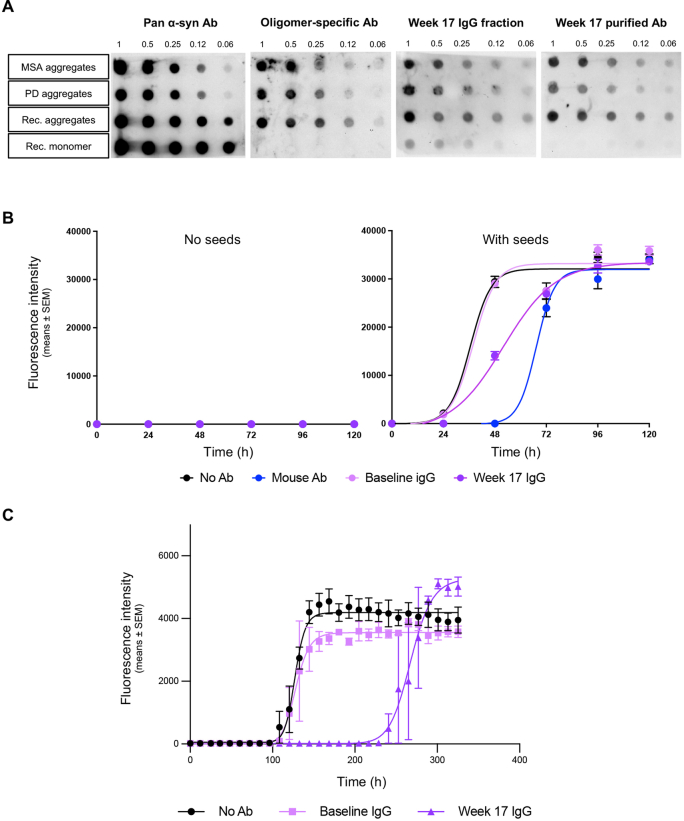
UB-312 derived antibodies preferentially bind aggregated αSyn and alter the kinetics of αSyn aggregation in the SAA. (**a**) Dot blot analyses indicate that IgG fractions and affinity purified antibodies collected from healthy volunteers post-immunization (week 17) preferentially bind aggregates of αSyn derived from patients with MSA or PD, as well as to aggregates of recombinant αSyn. (**b**) Post-immunization IgG fractions spiked into a buffer containing seeds delay the aggregation of αSyn. Data are means ± SEM. Samples were run in triplicate (**c**) Post-immunization IgG fractions spiked into a CSF sample from a patient with PD delay the aggregation of αSyn. Data are means ± SEM. Samples were run in duplicate. αSyn-SAA, alpha-synuclein seed amplification assay; Ab, antibody; CSF, cerebrospinal fluid; IgG, immunoglobulin G; MSA, multiple system atrophy; SEM, standard error of the mean; PD, Parkinson’s disease.

Postimmunization IgG fractions and affinity purified antibodies isolated from sera of healthy volunteers collected in Part A of this study demonstrated strong binding to aggregated αSyn (MSA-derived, PD-derived and recombinant-derived aggregates) and low binding to recombinant monomeric αSyn (Extended Data Fig. 1a). Furthermore, when spiked into a saline solution containing αSyn oligomers or into a CSF sample from a patient with PD, postimmunization IgG fractions readily altered the kinetics of αSyn aggregation (Extended Data Fig. 1b,c).

**Fig. 3:**
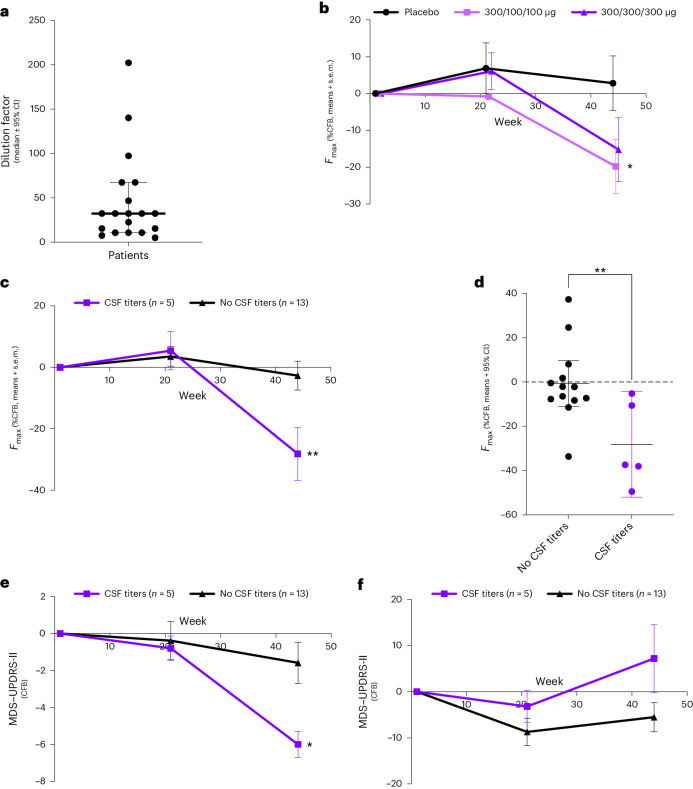
Evaluation of αSyn seeds in CSF and MDS–UPDRS scores in patients with PD with and without CSF titers. **a**, CSF samples collected before treatment (baseline) were serially diluted and tested in the αSyn-SAA (*n* = 20). Data presented are maximal dilution factor achieved before losing the signal in the αSyn-SAA; the bar represents median ± 95% CI. **b**, Maximum fluorescence (%CFB, mean ± s.e.m.) in CSF samples collected at baseline, week 21 and week 45 in patients treated with placebo (*n* = 6), UB-312 300/100/100 μg (*n* = 6, one patient provided CSF only at baseline and is excluded) or UB-312 300/300/300 μg (*n* = 6, one individual who was αSyn-SAA negative at baseline is excluded) showed a significant difference between placebo and 300/100/100 μg (two-way ANOVA, time × treatment interaction, *P* = 0.0343). **c**, A comparison of the maximum fluorescence (%CFB, mean ± s.e.m.) in CSF samples collected at baseline, week 21 and week 45 showed a significant difference (two-way ANOVA, time × treatment interaction, *P* = 0.0037) between individuals with (*n* = 5) versus without (*n* = 13) detectable antibody titers. **d**, Maximum fluorescence at EoS (%CFB means of three technical replicates per individual; bar represents median ± 95% CI) shows a significant between-group difference (two-tailed unpaired *t*-test, *P* = 0.0094). **e**, A comparison in CFB (mean ± s.e.m.) in MDS–UPDRS-II score showed a significant difference (unpaired *t*-test, time × group interaction, *P* = 0.016) between patients with (*n* = 5) versus without (*n* = 13) detectable CSF antibody titers. The subcomponent that showed the greatest difference in this change was ‘Getting out of bed, getting out of a car or standing up from a low chair’ (Supplementary Table 1). A two-way ANOVA with a mixed-effect model was followed by within-group analysis per Benjamini, Krieger and Yekitieli. **f**, A comparison in the CFB (mean ± s.e.m.) in MDS–UPDRS-III score between patients with (*n* = 5) versus without (*n* = 13) detectable CSF antibody titers. A two-way ANOVA was used as per **e**. In **a**–**f**, *P < 0.05 and **P < 0.01. CI, confidence interval.

In Part B, CSF samples were collected at weeks 1 (baseline), 21 and 45, except one patient in the 300/100/100 μg group who provided CSF only at baseline, and another only at baseline and week 21. Nineteen out of 20 baseline CSF samples tested positive in the Amprion αSyn-SAA. Excluding the negative sample, the median dilution factor was 32.40 (range 197.1) (Fig. 3a), indicating for subsequent analyses an optimal dilution of 1:5 to retain positivity status and prevent natural inhibitors such as lipoproteins from interfering with the αSyn-SAA seeding kinetics^21^. Individual seed amplification curves obtained at baseline and end of study (EoS) are illustrated in Supplementary Figs. 3–5. The patient who provided CSF only at baseline and the patient who was negative for αSyn-SAA were not included in the final analysis.

As illustrated in Fig. 3b, the maximum fluorescence (*F*_max_) assessed longitudinally indicated a significant change from baseline (CFB) (*F* = 6.622 (1.541–22.35), *P* = 0.009), with placebo showing a nonsignificant 2.8% increase, UB-312 (300/100/100 μg) showing a significant 19.8% decrease (*P* < 0.05) and UB-312 (300/300/300 μg) showing a nonsignificant 15.2% decrease at week 45. At week 45, *F*_max_ was significantly lower in patients treated with UB-312 300/100/100 μg versus placebo (*P* < 0.05). Interestingly, from a qualitative standpoint, one patient in the 300/300/300 μg group was αSyn-SAA positive at baseline and end of treatment, but αSyn-SAA negative at the end of study.

### Post hoc analyses

**Fig. 4:**
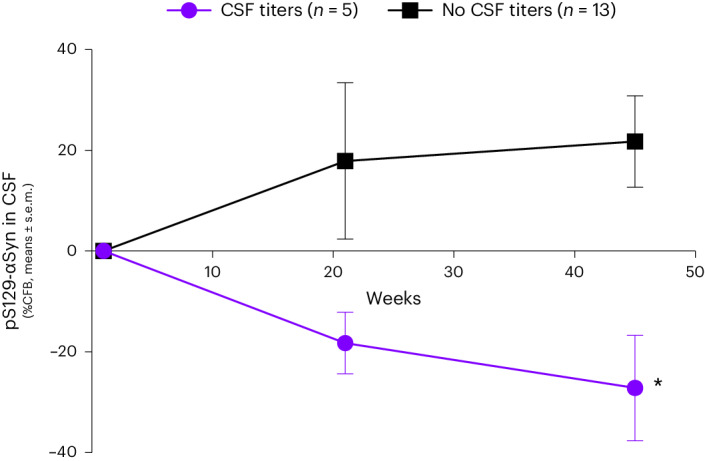
Levels of pS129-αSyn in the CSF of patients with detectable CSF antibodies after immunization with UB-312. CSF samples were analyzed in duplicate using immunomagnetic reduction as described in the methods, in patients with (*n* = 5) and without (*n* = 13) detectable CSF antibody titers. Data are expressed as percent CFB, mean ± s.e.m. A Bonferroni multiple comparisons test confirmed a significant difference between groups at EoS, *P = 0.0154.

An unplanned post hoc analysis to evaluate whether the reduction of *F*_max_ was related to CSF antibody titers (Fig. 3c,d) showed it was indeed more pronounced and statistically significant in individuals with detectable CSF antibody titers (as measured at week 21, *n* = 5) than those without (*n* = 13) at week 21 (time effect: *F* = 12.77 (1.73–26.82), *P* = 0.0002; treatment × time effect: *F* = 6.755 (2–31), *P* = 0.0037). Interestingly, a significant difference in CSF levels of pS129-αSyn was also observed between patients with or without detectable CSF antibodies (Fig. 4), further supporting an effect of UB-312 on pathological αSyn. Similarly, while there was no significant difference between treatment groups on the MDS–UPDRS-II and MDS–UPDRS-III (Supplementary Fig. 2), CFB on the MDS–UPDRS-II scale indicated a statistically significant improvement in individuals with detectable CSF antibody titers compared with other patients (*F* = 12.94 (1.569–24.32), *P* = 0.0004; treatment × time effect: *F* = 4.739 (2–31), *P* = 0.016; Fig. 3e,f).

## Discussion

This phase 1 Part B trial in patients with PD met its primary prespecified outcomes and showed that UB-312 was generally safe and well tolerated, and generated a time-dependent increase in anti-αSyn antibodies in both serum and CSF in patients with PD. No substantial differences in TEAEs were observed between the UB-312 groups and the placebo group, with most TEAEs being transient and self-resolving.

In healthy volunteers, UB-312 was generally safe and well tolerated up to a dose regimen of 300/300/300 μg without SAEs^19^. In the higher dose regimens in healthy volunteers, influenza-like symptoms were observed in two participants^19^. These events were not observed in patients with PD. The frequency and intensity of TEAEs were comparable between healthy volunteers and patients with PD, and similar TEAEs seemed to occur in both populations (headache, lumbar puncture site pain and fatigue). Local injection-site reactions did not increase after subsequent administrations, and no severe local reactions were observed (as seen previously with other active immunotherapies targeting αSyn)^7,8,10,22^. One participant reported a deep venous thrombosis 50 days after administration of a second dose of UB-312 for which no etiological cause could be found, and which was therefore considered possibly related to study drug. Nevertheless, this venous thrombo-embolism does not follow the venous thrombo-embolism events observed after other vaccinations and therefore may not be related^23–25^. Further research should investigate the potential relationship.

In both healthy volunteers and patients with PD, UB-312 vaccination generated similar time-dependent serum antibodies against the C-terminal epitope. In healthy volunteers, antibody levels were similar to those achieved in other active and passive immunization studies (that is, typically requiring serum antibody concentrations of ~10–20 μg ml^−1^ for target engagement)^7,19^. The levels were lower in patients with PD. Potentially, this difference could be attributed to comorbidities^26,27^, but a more likely cause may be that redistribution of antibodies to pathological tissues and target-mediated clearance differs in patients with PD compared with healthy volunteers, which has been demonstrated in preclinical studies^18,28^. That is, UB-312 does not induce antibodies against normal αSyn. Higher levels of UB-312-induced antibodies in healthy volunteers could therefore result from lack of target-mediated clearance in this population, in comparison with patients with PD in whom aggregated αSyn is present in both peripheral and central nervous system tissues. The elicited antibodies could conceivably be sequestered in pathological peripheral tissues after leaving the blood. Another possibility is that patients with PD may have a compromised immune system less able to respond to immunization^29^, which is supported by a recent report suggesting that B cell numbers in peripheral blood are reduced in patients with PD^30^. This potential alteration in immune response will be investigated in future clinical development studies in patients with PD.

Dosing regimens are less straightforward for active compared with passive immunotherapies. Rather than escalating based solely on total amount of drug product administered over time, factors including time between administrations and the relationship between prime-boost doses can also be varied. The dose–response observed in Part A suggested that a low prime dose followed by high boost doses may be less immunogenic^19^. In Part B, the two selected regimens had the same high prime dose, followed by two different boost dose regimens. Interestingly, the high prime dose followed by lower boost doses seemed to be more immunogenic, albeit not statistically significant. Clearly, further investigations of different doses and additional boosts will be required to better understand the pharmacological response to an active immunization against endogenous proteins.

We also investigated the use of αSyn-SAA. Originally developed to detect small amounts of pathological misfolded proteins in biological samples^20^, αSyn-SAA leverages the intrinsic self-replicative nature of misfolded αSyn aggregates (αSyn seeds), which can initiate polymerization of monomeric protein. Study samples are combined with excess monomeric αSyn and subjected to cycles of fragmentation and elongation, which can amplify the biomarker to detectable levels^31^. αSyn-SAA has demonstrated excellent sensitivity and specificity for detection of pathological forms of αSyn in CSF from patients with PD^32–34^, can discriminate CSF samples from patients with PD, patients with MSA and healthy individuals^35^, and is becoming progressively accepted as a supporting tool for diagnosis of PD.

To our knowledge, this is the first report showing a positive effect of an investigational therapy on the αSyn-SAA signal. UB-312-induced antibodies, purified from immunized healthy volunteers, preferentially bound aggregated αSyn and delayed the seeding kinetics of αSyn whether in a CSF sample from a patient with PD or in a solution containing recombinant aggregates of αSyn. We found that active immunization with UB-312 was associated with reductions in the αSyn-SAA signal in CSF as measured by *F*_max_, which can be interpreted as a sign of in vivo target engagement. The reduction indicates that the αSyn seeds are not recruiting the whole pool of αSyn monomers that were spiked in, and could be the result of reduced number of αSyn seeds, type of seeds and/or a reduction of a specific pool of seeds more prone to aggregate. The *F*_max_ readings were stable between triplicate measurements from individual patients (Supplementary Figs. 3–5). Interestingly, one patient who had αSyn-SAA positivity at baseline no longer did at end of study, after treatment with UB-312 300/300/300 μg. Moreover, that the MDS–UPDRS-II scores also improved in patients with CSF antibody titers, in line with the change in αSyn-SAA signal, is intriguing, though anecdotal given the small cohort size. A much larger trial will be required to show clinical efficacy. It is also important to note that αSyn-SAA has not yet been validated as a quantitative measure and the rate of conversion from a reduction in *F*_max_ to reduction in misfolded synuclein is unknown as fluorescence is measured in arbitrary units. However, in the absence of a valid biomarker of target engagement, these data suggest a potential route to quantifiable assessment of an effect of treatment on pathology in future clinical trials, using an assay that is already increasingly deployed in a diagnostic setting.

There are several limitations to this study. First, the sample size was small; 13 patients with PD completed vaccination with UB-312. However, it is not an abnormally low number for a first-in-patient study^36,37^. Second, demographic characteristics were comparable between groups, and all patients were on stable medication for PD before enrolling. Nevertheless, as the study aimed to determine antibody responses to vaccination, this relatively homogeneous population was deemed suitable. Patients were not screened for αSyn-SAA positivity or genetic predispositions, biomarkers that might facilitate selection of a relevant patient population for αSyn-targeted therapies. We also did not evaluate lipoprotein levels in the CSF to confirm stability and lack of interference with αSyn aggregation^21^; however, the dilution factor of 1:5 was sufficient to avoid this effect, and it is unlikely that any CSF constituents could have influenced the signal only at the end of the study. Additionally, CSF antibodies could be detected using a titer assay; however, CSF antibody concentrations were all below the lower limit of quantification. A difference in processing or analysis method could potentially contribute to this discrepancy between the two assays. Either way, our results suggest that antibodies in CSF must reach detectable levels to have an effect on αSyn-SAA. Future dose optimization will be needed to achieve greater CSF antibody titer exposures and to confirm the effects of UB-312 on αSyn-SAA.

UB-312 is not the only immunotherapy targeting αSyn to enter clinical development in PD; clinical data have been published for active and passive immunotherapies^8,11–13^. Compared with passive immunization therapy, which typically requires frequent administration at high costs, vaccination is likely to offer greater accessibility and convenience, requiring administration only every 3–12 months after the priming regimen to sustain an antibody response^38^. This minimized burden of administration and visits to a clinic is particularly advantageous for patients with advanced PD. Regardless of approach, this study of UB-312, which is to be corroborated in future phases of clinical development, is the first to assess an immunotherapeutic effect on pathological forms of αSyn as measured by αSyn-SAA.

In conclusion, this first-in-patient trial of an active immunotherapeutic targeting aggregated αSyn in patients with PD met its primary outcomes of safety, tolerability and immunogenicity. UB-312 was observed to safely overcome immune tolerance, inducing antibodies specific against pathological forms of αSyn and importantly able to cross the blood–brain barrier. The results are consistent with conclusions from Part A of the phase 1 study and with preclinical studies. Together, these data support continued development of UB-312 as a disease-modifying treatment for PD. Future trials should focus on optimizing dose and antibody exposure in CSF over longer treatment periods, and further assess the safety and efficacy of UB-312 in synucleinopathies.

## Methods

### Study design

This was a phase 1, single-center, randomized, double-blind, placebo-controlled clinical trial (Part B) conducted at the Centre for Human Drug Research (CHDR), the Netherlands, in accordance with the Declaration of Helsinki and International Council for Harmonisation Good Clinical Practice guidelines. Independent ethics approval for the protocol was granted by the Beoordeling Ethiek Biomedisch Onderzoek, Assen, the Netherlands, and all participants provided written informed consent. Participants received an allowance for participation and were reimbursed for travel expenses. There were no important protocol changes after trial commencement. Samples from the healthy volunteer cohort from Part A of the study (methods previously published^19^) were used in biomarker analyses. Study details are available at ClinicalTrials.gov, NCT04075318.

### Participants

Recruitment to Part B used an existing database and advertisements. Males (*n* = 16) and females (*n* = 4) (sex/gender was determined based on self-report and collected on the case report form) with a diagnosis of PD (as confirmed by a treating general practitioner or neurologist, and including dopaminergic deficiency (see below)), H&Y Stage ≤III at screening, aged 40–85 years, with a body mass index (BMI) of 18–32 kg m^−2^, who were postmenopausal, surgically sterile or using adequate contraception, with no clinical abnormalities based on medical history, physical examination, clinical laboratory evaluations and 12-lead ECGs were eligible if they were expected to be able to undergo all study procedures. As part of the screening, a baseline MRI was performed to exclude patients with structural brain abnormalities, and a dopamine transporter (DaT) scan was performed if no historic DaT scan was available to confirm the loss of dopaminergic activity as part of the PD diagnosis. Participants were allowed to use concomitant medication for PD or other comorbidities if the regimen was stable before first injection (from 30 days before first study drug administration for permitted antiparkinsonian medications or 60 days for monoamine oxidase B inhibitor (MAO-B) inhibitors), and expected to remain stable throughout the study. An unscheduled MRI could be requested per the investigator’s judgment for safety evaluation. Participants were excluded if they had clinical abnormalities or history of medical, neurological or psychiatric conditions that the investigator judged might compromise their safety or scientific value of the study; acute or chronic infection with human immunodeficiency virus, hepatitis C virus or hepatitis B virus; history or evidence of an autoimmune disorder; history of anergy; and history of allergy or any confirmed allergic reactions. Participants were also ineligible if they had participated in any clinical trial with monoclonal antibodies or vaccines directed at αSyn, had any other known or suspected cause of Parkinsonism besides idiopathic PD; had history or evidence at screening of PD-related freezing episodes, falls or orthostatic hypotension; had a DaT scan inconsistent with dopamine transporter deficit; or had any neurological disease other than PD.

### Randomization and blinding

Eligible participants were randomized by code (SAS version 9.4) to UB-312 or placebo within treatment cohort 1 (300/100/100 μg or placebo), cohort 2 (300/300/300 μg or placebo) by an independent statistician, without restrictions or stratifications, in consecutive order and numbered according to treatment cohort. Both cohorts consisted of ten participants and were randomized 7:3 (UB-312:placebo). Individual randomization codes were placed in a single sealed envelope, labeled ‘emergency decoding envelopes’ and kept in a safe cabinet at the clinical site.

Syringes with either UB-312 or placebo were prepared by an independent, unblinded pharmacist at the Leiden University Medical Centre. Both had an identical white, opaque appearance. Participants and clinical staff at the site were blinded to the treatment during the clinical conduct of the study.

### Procedures

UB-312 or placebo was administered intramuscularly in the deltoid muscle on weeks 1, 5 and 13, and return visits were planned on weeks 2, 6, 9, 14, 21 (considered end of treatment), 29, 37 and 45 (considered EoS). A review of concomitant medication, TEAEs and vital signs were done at every visit. Physical and neurological assessments were done at weeks 1, 5, 13, 21, 29, 37 and 45. A triplicate of ECG was done at weeks 1, 5 and 13 (both 45 min preadministration, as well as 6 h postadministration) and week 21. Safety blood and urine tests including full blood count, coagulation, electrolytes, liver and renal function, C-reactive protein and erythrocyte sedimentation rate were assessed at weeks 1, 5, 13, 17 and 21, and if applicable, all results were available before the administration. A pregnancy test was done at weeks 1, 5 and 13 (all preadministration). A urine drug test and alcohol breath test were done at week 1 only, preadministration. A safety telephone contact was conducted the day after each administration of UB-312 or placebo.

CSF was sampled at weeks 1 (preadministration), 21 and 45, and was used both to assess safety as well as free and total αSyn and anti-αSyn antibodies. CSF was collected using CHDR standard operating procedures, processed on ice, analyzed for white and red blood cell counts, protein, glucose, albumin and hemoglobin within 2 h of collection, and discarded if red blood cells were present. Within 60 min of collection, CSF samples were aliquoted and frozen immediately on dry ice, and then stored upright at −80 °C. Antibodies against FL αSyn and against the C-terminal epitope, αSyn antibodies against components of the vaccine (CpG1 and the T-helper peptide) and free and total αSyn in blood were sampled at every study visit. Cytokines in blood were sampled at weeks 1, 5, 13 and 21. T cell enzyme-linked immunosorbent spot in peripheral blood mononuclear cells was sampled at weeks 1 and 17. Human leukocyte antigen in blood was sampled at week 1.

The MoCA and the MDS–UPDRS parts II and III, including H&Y classification, were done at weeks 1 (preadministration), 21 and 45. An individual H&Y classification was also conducted at screening. All MDS–UPDRS assessments were performed in the ON state. Before the actual assessments, the assessor confirmed verbally with the patient if the patient was indeed in the ON state.

Participants were provided with a paper diary for self-recording of solicited local vaccination-site reactions (that is, pain, tenderness, erythema/redness and induration/swelling) and systemic reactions (that is, fever, nausea/vomiting, diarrhea, headache, fatigue, myalgia and illness) during a 7-day period after each administration.

### Outcomes

The primary endpoints were to evaluate the safety and tolerability as determined by the assessment of TEAEs, safety blood and urine tests, neurological and physical examinations, ECG and immunogenicity as determined by anti-αSyn antibodies in blood and CSF. The exploratory objectives for the study including Part A were to determine the immunogenicity of UB-312 against components of the vaccine, and differences in total αSyn and free αSyn in blood and CSF, while exploratory outcomes specific to Part B comprised effects on MDS–UPDRS and MoCA, and target engagement by αSyn-SAA. Bioanalytical and biomarker methods were previously fully described^19^.

### Antibody purification

Sera collected pre- or postimmunization from the healthy volunteer cohort were pooled using 200 μl per sample. Protein A plus spin columns (Thermo Fisher, 89960) were equilibrated with protein A IgG binding buffer (Thermo Fisher, 21001) at room temperature followed by centrifugation at 1,000*g* for 1 min. Sera samples were applied to the column and incubated at room temperature with end-over-end mixing for 10 min. Columns were then placed in a new 50 ml collection tube and centrifuged for 1 min at 1,000*g*. Columns were then washed 3× by adding 10 ml of binding buffer and centrifuged for 1 min. Next, 500 μl of neutralization buffer (Thermo Fisher, 89948) was added to three 50 ml collection tubes. Columns were then placed into one of the collection tubes and 5 ml of elution buffer (Thermo Fisher, 21004) was added to the column and centrifuged for 1 min into the first of the three collection tubes with neutralization buffer. Spin columns were transferred to another tube that contains neutralization buffer, saving the collected solution as the first elution fraction. These steps were repeated to obtain three fractions. Pooled IgG fractions were then buffer exchanged and concentrated using an Amicron Ultra-15 centrifugal filter 50 kDa molecular weight cutoff (Millipore Sigma, UFC905024) per the manufacturer’s instructions. For affinity purification, epitope-specific peptide-linked columns were washed with wash buffer 3× at room temperature. Sample IgG fractions were then added and incubated at room temperature, gently mixing end-over-end. Immunodepleted samples were then collected and set aside to evaluate potential residual binding efficiency. The column was then washed 6× with 2 ml of wash buffer. Samples were eluted by centrifuging into a clean tube with neutralization solution 5× with 2 ml of elution buffer. Affinity purified antibodies were then buffer exchanged as described above for IgG fractions.

### SAA

The αSyn-SAA methodology was performed according to Shahnawaz et al.^34^. Briefly, human CSF samples (40 µl) were added to the wells of an opaque 96-well plate (Costar, 3916). Thereafter, seed-free αSyn at a concentration of 1 mg ml^−1^ in 100 mM piperazine-N,N′-bis(2-ethanesulfonic acid (PIPES), pH 6.5 and 500 mM NaCl was added to each well in the presence of 5 μM of thioflavin T at a final volume of 200 μl. Samples were subjected to cyclic agitation (1 min at 500 r.p.m. followed by 29 min without shaking) at 37 °C on a SpectraMax Gemini EM Microplate Reader (Molecular Devices). The increase in thioflavin T fluorescence was monitored at excitation of 435 nm and emission of 485 nm periodically.

To determine the optimal dilution across samples and for the evaluation of the kinetic parameters after UB-312 immunization, SAA was performed as described in Concha-Marambio et al.^31^, with modifications. The assay included 40 µl of sample and 60 µl of reaction mixture for a final 100 µl reaction comprising 0.3 mg ml^−1^ of recombinant αSyn (Amprion, S2020), 500 mM NaCl, 100 mM PIPES pH 6.5, 0.1% sarkosyl and 2 1/8’ silicone nitride beads (Tsubaki Nakashima). To assess the optimal dilution, CSF samples were threefold serially diluted in synthetic CSF (Amprion, S2022) up to 1:729 and evaluated in the assay. For the assessment of αSyn-SAA kinetics, CSF samples underwent a single fivefold dilution in synthetic CSF and were tested in triplicate.

### Dot blot analyses

First, 2 µl of purified αSyn protein either from recombinant or patient-derived preparations were spotted onto nitrocellulose membranes (Amersham Biosciences) and air dried for 30 min at room temperature. Patient-derived preparations were obtained from CSF samples that were submitted to two rounds of amplification in the αSyn-SAA, as described above. Membranes were blocked with 5% w/v nonfat dry milk in Tris-buffered saline–Tween 20 (20 mM Tris, pH 7.2, 150 mM NaCl and 0.05% (v/v) Tween 20) at room temperature for 2 h. After blocking, the membranes were probed with either a pan anti-αSyn antibody (BD Bioscience, 610787), an oligomer-specific anti-αSyn antibody (clone MJFR-14-6-4-2, Abcam, ab209538), IgG fractions or affinity purified antibodies isolated from sera at week 17 postimmunization. Species-relevant horseradish peroxidase-conjugated secondary antibodies (1:5,000) were then applied and blots were visualized using the enhanced chemiluminescence plus western blotting detection kit (Amersham Biosciences).

### Measurements of pS129-αSyn in CSF samples

CSF samples were collected as described above. The concentrations of CSF pS129-αSyn were measured using the Phospho-a-Synuclein S129 kit from MagQu (MagQu, MF-PS1-0060) and immunomagnetic reduction (IMR). Before measurement, CSF samples were thawed on ice and reagents were brought to room temperature. CSF was first diluted 20 times with PBS. Thereafter, 60 ml of diluted CSF sample were added to 60 ml of IMR reagent for IMR analysis. Each sample was assessed in duplicates using an IMR analyzer (XacPro-S, MagQu).

### Statistical analysis

The sample size was considered adequate to characterize the safety, tolerability and dose–response profile of UB-312’s immunogenicity, based on data from Part A in healthy volunteers. The trial was not powered for statistical comparisons between regimens, and results presented for safety and immunogenicity analyses are descriptive.

No interim analysis was planned for Part B. An analysis of immunogenicity and selected safety data for Part B was performed when the last patient in Part B complete the end of treatment (week 21) visit. The study continued as planned. The study team remained blinded to the treatment of individual patients until the end of the study.

Safety and tolerability were analyzed based on the safety population, defined as all participants randomized and exposed to at least one vaccination, identical to the mITT population. Analyses of immunogenicity and pharmacodynamic endpoints were performed by treatment allocation based on the PP population (all participants who received all planned vaccinations, up to the point of a protocol violation, if applicable, fulfilled all entry criteria and had no critical or major protocol deviations). There were no critical or major protocol deviations.

Baseline data were described by summary statistics of the mITT and PP populations. Immunogenicity and pharmacodynamic endpoints included in the analysis were concentrations of anti-αSyn (FL and C-terminal epitope); anti-CpG1 and anti-T-helper peptide antibodies; inflammatory markers; T cell enzyme-linked immunosorbent spot assay results; free and total αSyn in CSF and blood; and the total scores for MoCA, MDS–UPDRS part II and part III.

For immunogenicity, the seroconversion rate was provided as the percentage of participants that had no measurable (under the lower limit of quantification) FL and C-terminal epitope-specific anti-αSyn antibody levels before the first vaccination and subsequently developed quantifiable antibodies after the first vaccination.

FL anti-αSyn antibody concentration data were provided by one individual laboratory (QPS). Data for the epitope-specific anti-αSyn antibodies were provided by two laboratories. QPS provided antibody concentrations in ng ml^−1^ and Vaxxinity Laboratories provided antibody titers in log-dilution factor, which are provided in the results.

For exploratory outcomes, changes in *F*_max_ (relative fluorescence units), MDS–UPDRS and MoCA were analyzed. To analyze *F*_max_, samples were run in triplicate and average values utilized. Percentage CFB was calculated per individual from their week 1 value. For MDS–UPDRS-II and MDS–UPDRS-III, CFB was calculated per individual as the difference in score from their week 1 value. MoCA was summarized. An unplanned post hoc analysis was performed to evaluate *F*_max_, pS129-αSyn, MDS–UPDRS-II and MDS–UPDRS-III differences in individuals with and without detectable CSF antibody titers. A two-way analysis of variance (ANOVA) with a mixed-effect model, due to one missing sample at week 45, was used with time and treatment as factors. A significant time effect (*P* < 0.05) was followed by a within-group analysis using the method of Benjamini, Krieger and Yekitieli. Unpaired *t*-tests were used to compare differences between groups at each time point. Data are presented as means ± s.e.m.

Safety and statistical programming were conducted with SAS 9.4 for Windows (SAS Institute Inc.). Exploratory biomarker analyses were conducted with GraphPad Prism 10.1.1 for macOS (GraphPad Software).

There was an independent medical monitor. There was no data monitoring committee. This trial is registered with ClinicalTrials.gov: NCT04075318.

### Reporting summary

Further information on research design is available in the Nature Portfolio Reporting Summary linked to this article.

## Online content

Any methods, additional references, Nature Portfolio reporting summaries, source data, extended data, supplementary information, acknowledgements, peer review information; details of author contributions and competing interests; and statements of data and code availability are available at 10.1038/s41591-024-03101-8.

## Supplementary information

Supplementary Information Supplementary methods, Table 1 and Figs. 1–5.

Reporting Summary

## Data Availability

Deidentified data and study protocols used in this publication will be made available to qualified researchers who provide a valid research question within the scope of the informed consent, and may be subject to a data use agreement. Requests will be responded to within 30 days. Please direct inquiries to the corresponding author (Jean-Cosme Dodart, jc@vaxxinity.com).

## References

[1] Poewe, W. et al. Parkinson disease. *Nat. Rev. Dis. Prim.* **3**, 17013 (2017). 28332488 10.1038/nrdp.2017.13

[2] Bartels, T., Choi, J. G. & Selkoe, D. J. α-Synuclein occurs physiologically as a helically folded tetramer that resists aggregation. *Nature* **477**, 107–110 (2011). 21841800 10.1038/nature10324 PMC3166366

[3] Valdinocci, D., Radford, R. A. W., Siow, S. M., Chung, R. S. & Pountney, D. L. Potential modes of intercellular α-synuclein transmission. *Int. J. Mol. Sci.* **18**, 469 (2017). 28241427 10.3390/ijms18020469 PMC5344001

[4] Cole, N. B. et al. Lipid droplet binding and oligomerization properties of the Parkinson’s disease protein α-synuclein. *J. Biol. Chem.* **277**, 6344–6652 (2002). 11744721 10.1074/jbc.M108414200

[5] Melki, R. How the shapes of seeds can influence pathology. *Neurobiol. Dis.* **109**, 201–208 (2018). 28363800 10.1016/j.nbd.2017.03.011

[6] Luk, K. C. et al. Intracerebral inoculation of pathological α-synuclein initiates a rapidly progressive neurodegenerative α-synucleinopathy in mice. *J. Exp. Med.* **209**, 975–986 (2012). 22508839 10.1084/jem.20112457 PMC3348112

[7] Schenk, D. B. et al. First-in-human assessment of PRX002, an anti-α-synuclein monoclonal antibody, in healthy volunteers. *Mov. Disord.* **32**, 211–218 (2017). 27886407 10.1002/mds.26878 PMC5324684

[8] Volc, D. et al. Safety and immunogenicity of the α-synuclein active immunotherapeutic PD01A in patients with Parkinson’s disease: a randomised, single-blinded, phase 1 trial. *Lancet Neurol.* **19**, 591–600 (2020). 32562684 10.1016/S1474-4422(20)30136-8

[9] Nimmo, J. T. et al. Amyloid-β and α-synuclein immunotherapy: from experimental studies to clinical trials. *Front. Neurosci.* **15**, 733857 (2021). 34539340 10.3389/fnins.2021.733857 PMC8441015

[10] Brys, M. et al. Randomized phase I clinical trial of anti-α-synuclein antibody BIIB054. *Mov. Disord.* **34**, 1154–1163 (2019). 31211448 10.1002/mds.27738 PMC6771554

[11] Poewe, W. et al. Safety and tolerability of active immunotherapy targeting α-synuclein with PD03A in patients with early Parkinson’s disease: a randomized, placebo-controlled, phase 1 study. *J. Parkinsons Dis.* **11**, 1079–1089 (2021). 34092654 10.3233/JPD-212594 PMC8461711

[12] Pagano, G. et al. Trial of prasinezumab in early-stage Parkinson’s disease. *N. Engl. J. Med.* **387**, 421–432 (2022). 35921451 10.1056/NEJMoa2202867

[13] Lang, A. E. et al. Trial of cinpanemab in early Parkinson’s disease. *N. Engl. J. Med.* **387**, 515–522 (2022). 10.1056/NEJMoa2203395 35921450

[14] Jensen, P. H., Schlossmacher, M. G. & Stefanis, L. Who ever said it would be easy? Reflecting on two clinical trials targeting α-synuclein. *Mov. Disord.* **38**, 378–384 (2023). 36645106 10.1002/mds.29318

[15] Chapleau, M., Iaccarino, L., Soleimani-Meigooni, D. & Rabinovici, G. D. The role of amyloid PET in imaging neurodegenerative disorders: a review. *J. Nucl. Med.* **63**, 13S–19S (2022). 35649652 10.2967/jnumed.121.263195 PMC9165727

[16] Wang, C. Y. et al. UB-311, a novel UBITh amyloid β peptide vaccine for mild Alzheimer’s disease. *Alzheimers Dement.* **3**, 262–272 (2017). 10.1016/j.trci.2017.03.005 PMC5651432 29067332

[17] Nimmo, J. T. et al. Novel antibodies detect additional α-synuclein pathology in synucleinopathies: potential development for immunotherapy. *Alzheimers Res. Ther.* **12**, 159 (2020). 33256825 10.1186/s13195-020-00727-x PMC7702704

[18] Nimmo, J. T. et al. Immunisation with UB-312 in the Thy1SNCA mouse prevents motor performance deficits and oligomeric α-synuclein accumulation in the brain and gut. *Acta Neuropathol.* **143**, 55–73 (2022). 34741635 10.1007/s00401-021-02381-5 PMC8732825

[19] Yu, H. J. et al. A randomized first-in-human study with UB-312, a UBITh α-synuclein peptide vaccine. *Mov. Disord.* **37**, 1416–1424 (2022). 35426173 10.1002/mds.29016 PMC9545051

[20] Saborio, G. P., Permanne, B. & Soto, C. Sensitive detection of pathological prion protein by cyclic amplification of protein misfolding. *Nature* **411**, 810–813 (2001). 11459061 10.1038/35081095

[21] Bellomo, G. et al. Cerebrospinal fluid lipoproteins inhibit α-synuclein aggregation by interacting with oligomeric species in seed amplification assays. *Mol. Neurodegener.* **18**, 20 (2023). 37005644 10.1186/s13024-023-00613-8 PMC10068178

[22] Kuchimanchi, M., Monine, M., Kandadi Muralidharan, K., Woodward, C. & Penner, N. Phase II dose selection for alpha synuclein-targeting antibody cinpanemab (biib054) based on target protein binding levels in the brain. *CPT Pharmacomet. Syst. Pharm.* **9**, 515–522 (2020). 10.1002/psp4.12538 PMC7499191 32613752

[23] Marietta, M., Coluccio, V. & Luppi, M. Potential mechanisms of vaccine-induced thrombosis. *Eur. J. Intern Med* **105**, 1–7 (2022). 35953336 10.1016/j.ejim.2022.08.002 PMC9359676

[24] Vickers, E. R. et al. Risk of venous thromboembolism following influenza vaccination in adults aged 50 years and older in the Vaccine Safety Datalink. *Vaccine* **35**, 5872–5877 (2017). 28888342 10.1016/j.vaccine.2017.08.086 PMC6508529

[25] Vallone, M. G. et al. Thrombotic events following COVID-19 vaccines compared to influenza vaccines. *Eur. J. Intern. Med.* **99**, 82–88 (2022). 35288031 10.1016/j.ejim.2022.03.002 PMC8904150

[26] Ciabattini, A. et al. Vaccination in the elderly: the challenge of immune changes with aging. *Semin. Immunol.* **40**, 83–94 (2018). 30501873 10.1016/j.smim.2018.10.010

[27] Spiegel, K. et al. A meta-analysis of the associations between insufficient sleep duration and antibody response to vaccination. *Curr. Biol.* **33**, 998–1005.e2 (2023). 36917932 10.1016/j.cub.2023.02.017

[28] Bae, E. J. et al. Antibody-aided clearance of extracellular α-synuclein prevents cell-to-cell aggregate transmission. *J. Neurosci.* **32**, 13454–13469 (2012). 23015436 10.1523/JNEUROSCI.1292-12.2012 PMC3752153

[29] Contaldi, E., Magistrelli, L. & Comi, C. in *Handbook of Clinical Neurology* vol. 193 67–93 (Elsevier, 2023). 10.1016/B978-0-323-85555-6.00008-4 36803824

[30] Zhang, Z. et al. Abnormal immune function of B lymphocyte in peripheral blood of Parkinson’s disease. *Parkinsonism Relat. Disord.* **116**, 105890 (2023). 37839276 10.1016/j.parkreldis.2023.105890

[31] Concha-Marambio, L., Pritzkow, S., Shahnawaz, M., Farris, C. M. & Soto, C. Seed amplification assay for the detection of pathologic alpha-synuclein aggregates in cerebrospinal fluid. *Nat. Protoc.* **18**, 1179–1196 (2023). 36653527 10.1038/s41596-022-00787-3 PMC10561622

[32] Siderowf, A. et al. Assessment of heterogeneity among participants in the Parkinson’s Progression Markers Initiative cohort using α-synuclein seed amplification: a cross-sectional study. *Lancet Neurol.* **22**, 407–417 (2023). 37059509 10.1016/S1474-4422(23)00109-6 PMC10627170

[33] Ning, H. et al. Baseline concentration of misfolded α-synuclein aggregates in cerebrospinal fluid predicts risk of cognitive decline in Parkinson’s disease. *Neuropathol. Appl. Neurobiol.* **45**, 398–409 (2019). 30346044 10.1111/nan.12524 PMC7380054

[34] Shahnawaz, M. et al. Development of a biochemical diagnosis of parkinson disease by detection of α-synuclein misfolded aggregates in cerebrospinal fluid. *JAMA Neurol.* **74**, 163–172 (2017). 27918765 10.1001/jamaneurol.2016.4547

[35] Shahnawaz, M. et al. Discriminating α-synuclein strains in Parkinson’s disease and multiple system atrophy. *Nature* **578**, 273–277 (2020). 32025029 10.1038/s41586-020-1984-7 PMC7066875

[36] Hazama, S. et al. A phase I study of combination vaccine treatment of five therapeutic epitope–peptides for metastatic colorectal cancer; safety, immunological response and clinical outcome. *J. Transl. Med.* **12**, 63 (2014). 24612787 10.1186/1479-5876-12-63 PMC4007571

[37] Aruga, A. et al. Phase I clinical trial of multiple-peptide vaccination for patients with advanced biliary tract cancer. *J. Transl. Med.* **12**, 61 (2014). 24606884 10.1186/1479-5876-12-61 PMC4015445

[38] Ovacik, M. & Lin, K. Tutorial on monoclonal antibody pharmacokinetics and its considerations in early development. *Clin. Transl. Sci.* **11**, 540–552 (2018). 29877608 10.1111/cts.12567 PMC6226118

