# Supplementary material for: Target engagement and immunogenicity of an active immunotherapeutic targeting pathological α-synuclein: a phase 1 placebo-controlled trial
Source: Nat Med. 2024 Jun 20;30(9):2631–40. doi: 10.1038/s41591-024-03101-8 (PMC11405261; doi:10.1038/s41591-024-03101-8)
Supplement: Supplementary file 1 — Supplementary methods, Table 1 and Figs. 1–5. [file 41591_2024_3101_MOESM1_ESM.pdf]

nature medicine

Supplementary information https://doi.org/10.1038/s41591-024-03101-8

Target engagement and immunogenicity of an active immunotherapeutic targeting pathological α-synuclein: a phase 1 placebo-controlled trial

In the format provided by the authors and unedited

Supplementary material

Table of contents

Supplementary Tables 2

Supplementary Table S1: Model-adjusted estimates of the mean differences between patients with and without CSF antibody titers in each item of the MDS-UPDRS part II score 2

Supplementary Figures 3

Supplementary Figure S1: MoCA scores from baseline to Week 45 per treatment group 3

Supplementary Figure S2: MDS-UPDRS scores from baseline to Week 45 per treatment group 4

Supplementary Figure S3: Individual αSyn aggregation curves in the αSyn-SAA at baseline and EoS in the placebo group 5

Supplementary Figure S4: Individual αSyn aggregation curves in the αSyn-SAA at baseline and EoS in the 300/100/100 μg group 6

Supplementary Figure S5: Individual αSyn aggregation curves in the αSyn-SAA at baseline and EoS in the 300/300/300 μg group 7

Supplementary Tables

Supplementary Table S1: Model-adjusted estimates of the mean differences between patients with and without CSF antibody titers in each item of the MDS-UPDRS part II score

Item	Estimate (95% CI)
Getting out of bed, getting out of a car, or standing up from a low chair	-0.62 (-1.15, -0.08)
Speech	-0.53 (-1.31, 0.24)
Saliva and drooling	-0.51 (-1.48, 0.47)
Walking and balance	-0.40 (-1.11, 0.30)
Hygiene	-0.28 (-0.62, 0.07)
Hobbies and other activities	-0.27 (-0.92, 0.38)
Handwriting	-0.21 (-1.50, 1.09)
Chewing and swallowing	-0.12 (-0.61, 0.37)
Turning in bed	-0.08 (-0.73, 0.57)
Getting dressed	-0.07 (-0.63, 0.48)
Tremor	0.04 (-0.48, 0.57)
Eating	0.11 (-0.53, 0.75)
Freezing*	NA
**MDS-UPDRS part II total**	**-2.96 (-5.90, -0.01)**

Estimates are adjusted mean differences in change from baseline to Week 45 between patients with (n=5) and without (n=13) detectable CSF antibody titers. The item showing the greatest difference in this change was “Getting out of bed, getting out of a car, or standing up from a low chair.” The model used MMRM to account for differences in the MDS-UPDRS part II between groups at baseline. Unadjusted changes from baseline in MDS-UPDRS part II total score are shown for patients with (n=5) and without (n=13) detectable CSF antibody titers in **Figure 3E**.

*For “Freezing” all responses were 0 so no estimate could be made.

CI, confidence interval; CSF, cerebrospinal fluid; MDS-UPDRS, Movement Disorder Society-Unified Parkinson’s Disease Rating Scale; MMRM, Mixed Model Repeated Measures.

Supplementary Figures

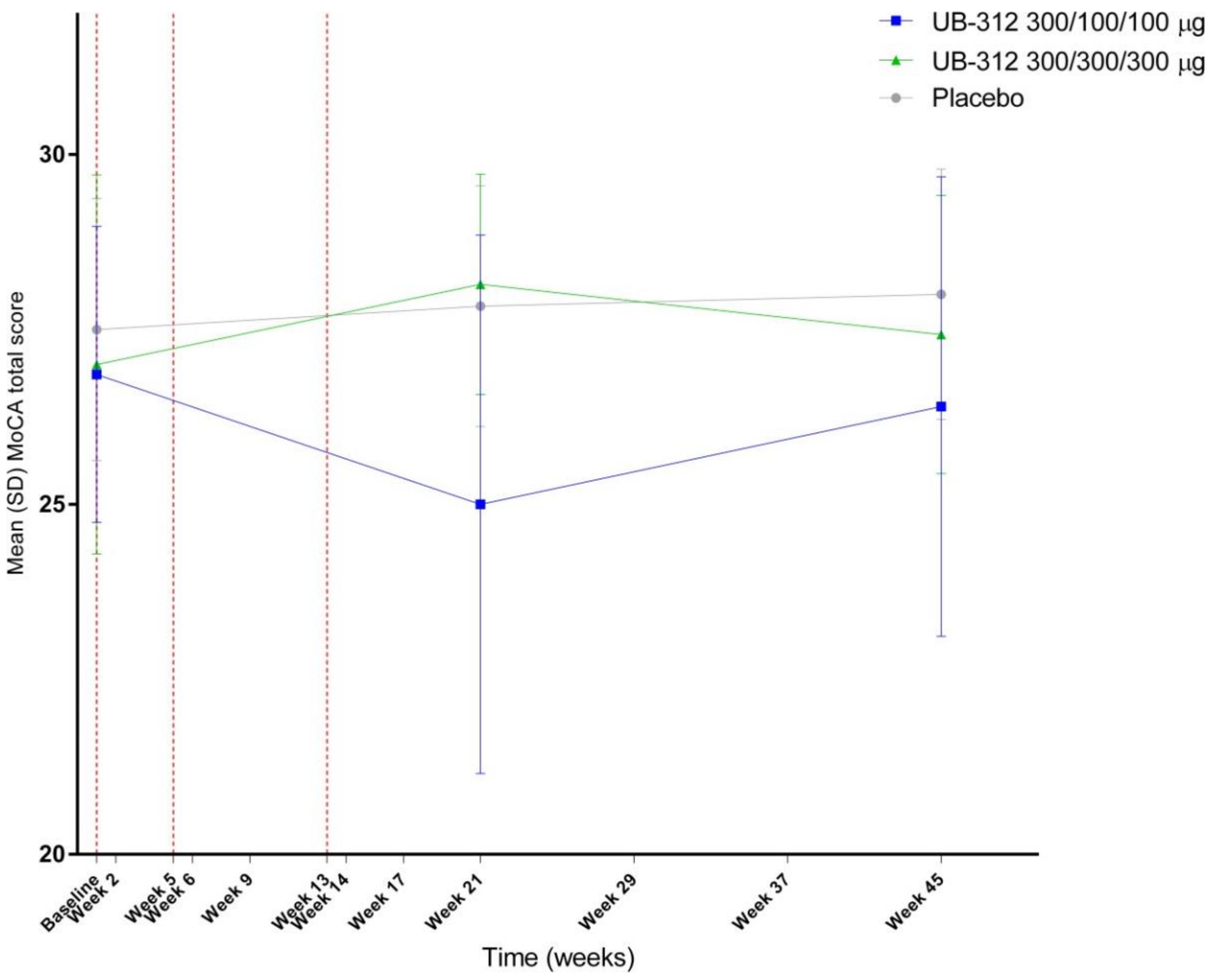

Supplementary Figure S1: MoCA scores from baseline to Week 45 per treatment group
Symbols represent mean (SD) MoCA total scores for each group at each timepoint. Timing of the administration of UB-312 or placebo has been depicted as red dotted lines. Numbers per group; placebo, n=6; 300/100/100 µg, n=7 up to Week 13, n=6 up to Week 45, and n=5 at Week 45; 300/300/300 µg, n=7.

MoCA, Montreal Cognitive Assessment; SD, standard deviation.

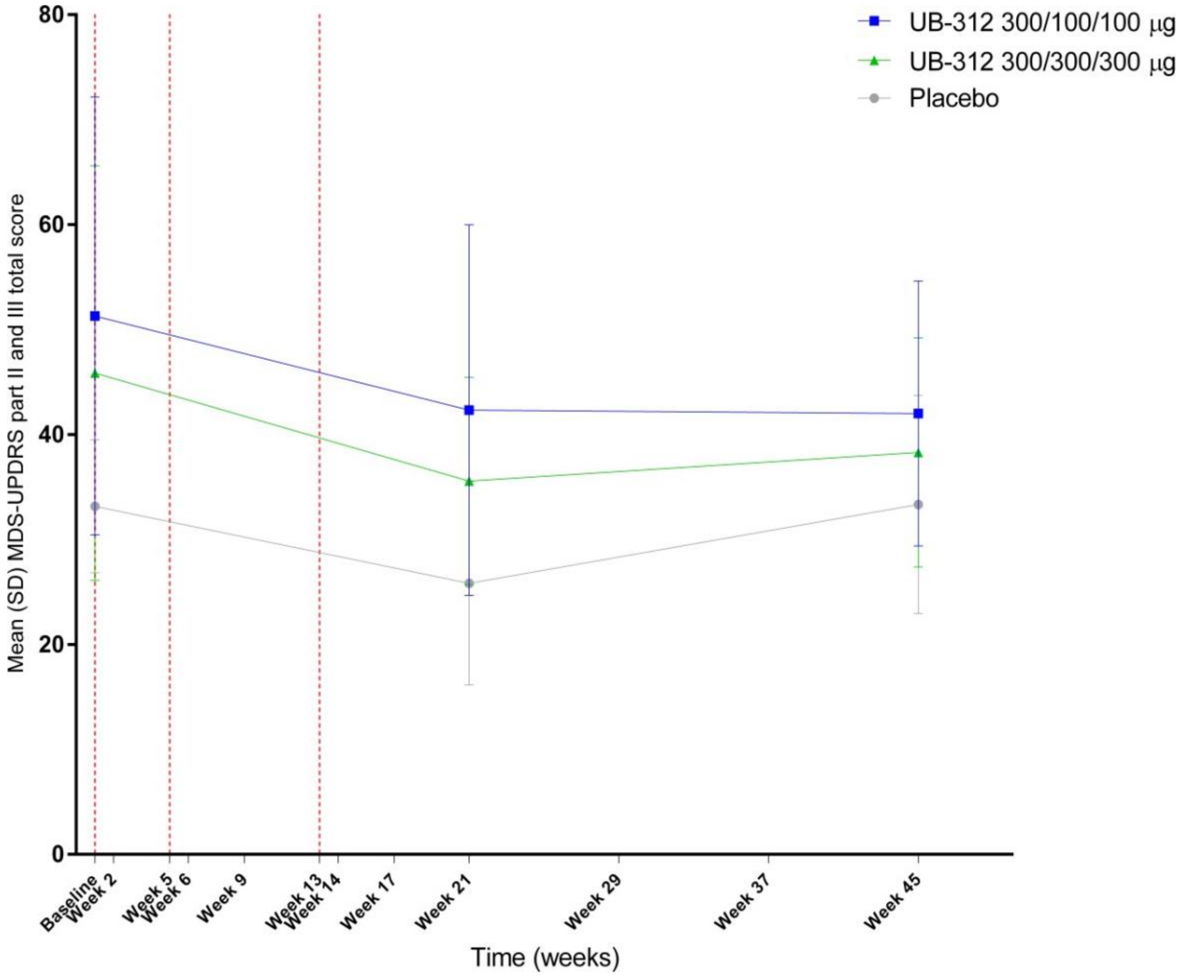

Supplementary Figure S2: MDS-UPDRS scores from baseline to Week 45 per treatment group

Symbols represent mean (SD) MDS-UPDRS part II and III total scores for each group at each timepoint. Timing of the administration of UB-312 or placebo has been depicted as red dotted lines. Numbers per group; placebo, n=6; 300/100/100 µg, n=7 up to Week 13, n=6 up to Week 45, and n=5 at Week 45; 300/300/300 µg, n=7.

MDS-UPDRS, Movement Disorder Society-Unified Parkinson’s Disease Rating Scale; SD, standard deviation.

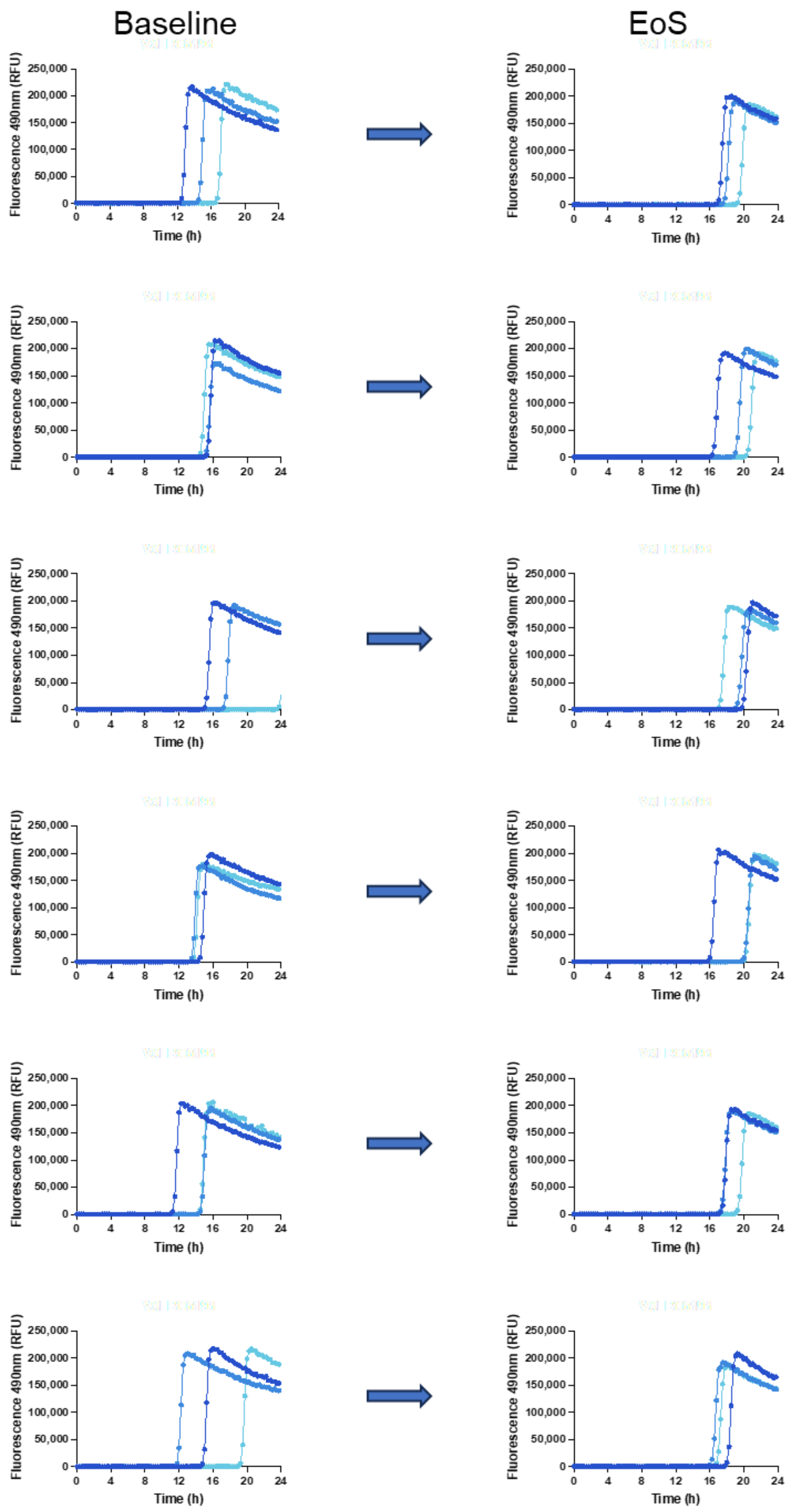

Supplementary Figure S3: Individual αSyn aggregation curves in the αSyn-SAA at baseline and EoS in the placebo group

Each graph represents the seeding kinetics of each triplicate from one CSF sample (i.e., one individual).

αSyn-SAA, alpha-synuclein seed amplification assay; CSF, cerebrospinal fluid; EoS, end of study

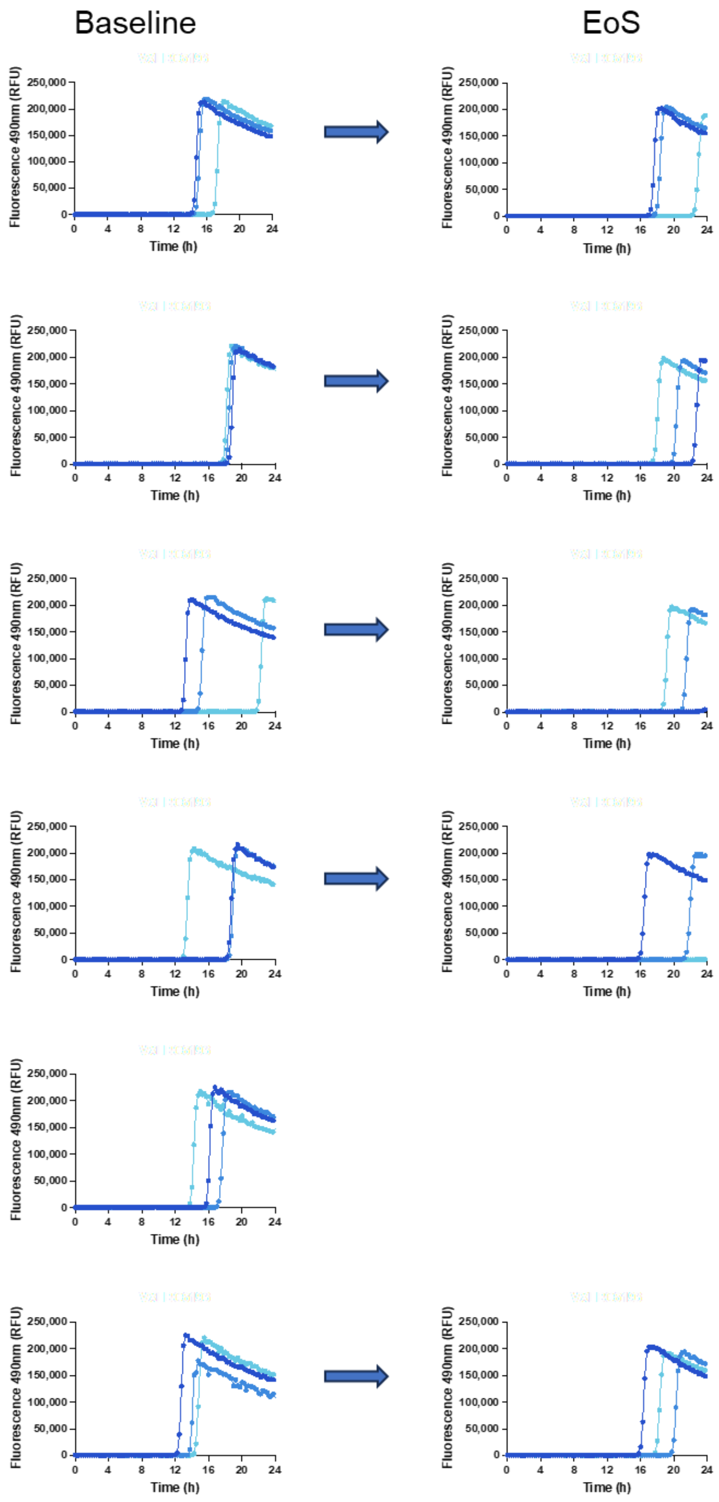

Supplementary Figure S4: Individual αSyn aggregation curves in the αSyn-SAA at baseline and EoS in the 300/100/100 μg group

Each graph represents the seeding kinetics of each triplicate from one CSF sample (i.e., one individual). One patient with αSyn-SAA positivity at baseline was no longer αSyn-SAA positive at EoS.

αSyn-SAA, alpha-synuclein seed amplification assay; CSF, cerebrospinal fluid; EoS, end of study

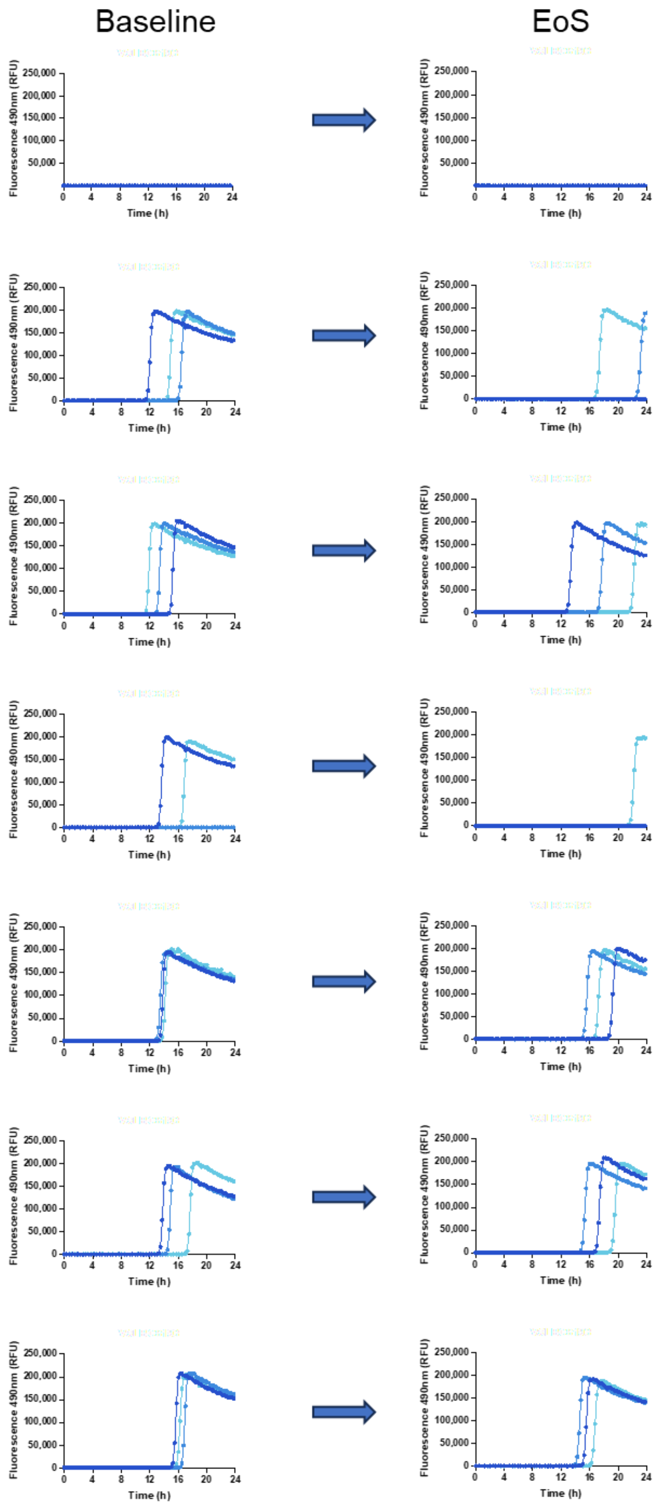

Supplementary Figure S5: Individual αSyn aggregation curves in the αSyn-SAA at baseline and EoS in the 300/300/300 μg group

Note that one individual did not provide CSF at EoS and no data could be collected at that timepoint for this individual. Each graph represents the seeding kinetics of each triplicate from one CSF sample (i.e., one individual).

αSyn-SAA, alpha-synuclein seed amplification assay; CSF, cerebrospinal fluid; EoS, end of study
