# Supplementary material for: Target engagement and immunogenicity of an active immunotherapeutic targeting pathological α-synuclein: a phase 1 placebo-controlled trial
Source: Nat Med. 2024 Jun 20;30(9):2631–40. doi: 10.1038/s41591-024-03101-8 (PMC11405261; doi:10.1038/s41591-024-03101-8)
Supplement: Supplementary file 2 — Reporting Summary [file 41591_2024_3101_MOESM2_ESM.pdf]

nature portfolio

Corresponding author(s): Jean-Cosme Dodart

Last updated by author(s): May 16, 2024

Reporting Summary

Nature Portfolio wishes to improve the reproducibility of the work that we publish. This form provides structure for consistency and transparency in reporting. For further information on Nature Portfolio policies, see our Editorial Policies and the Editorial Policy Checklist.

Statistics

For all statistical analyses, confirm that the following items are present in the figure legend, table legend, main text, or Methods section.

n/a	Confirmed	
☐	☒	The exact sample size (*n*) for each experimental group/condition, given as a discrete number and unit of measurement
☐	☒	A statement on whether measurements were taken from distinct samples or whether the same sample was measured repeatedly
☐	☒	The statistical test(s) used AND whether they are one- or two-sided<br>*Only common tests should be described solely by name; describe more complex techniques in the Methods section.*
☒	☐	A description of all covariates tested
☒	☐	A description of any assumptions or corrections, such as tests of normality and adjustment for multiple comparisons
☐	☒	A full description of the statistical parameters including central tendency (e.g. means) or other basic estimates (e.g. regression coefficient) AND variation (e.g. standard deviation) or associated estimates of uncertainty (e.g. confidence intervals)
☐	☒	For null hypothesis testing, the test statistic (e.g. *F*, *t*, *r*) with confidence intervals, effect sizes, degrees of freedom and *P* value noted<br>*Give P values as exact values whenever suitable.*
☒	☐	For Bayesian analysis, information on the choice of priors and Markov chain Monte Carlo settings
☒	☐	For hierarchical and complex designs, identification of the appropriate level for tests and full reporting of outcomes
☒	☐	Estimates of effect sizes (e.g. Cohen's *d*, Pearson's *r*), indicating how they were calculated

Our web collection on statistics for biologists contains articles on many of the points above.

Software and code

Policy information about availability of computer code

Data collection	No code used to collect data
Data analysis	Standard statistical package from SA 9·4, GraphPad Prism 10

For manuscripts utilizing custom algorithms or software that are central to the research but not yet described in published literature, software must be made available to editors and reviewers. We strongly encourage code deposition in a community repository (e.g. GitHub). See the Nature Portfolio guidelines for submitting code & software for further information.

Data

Policy information about availability of data

All manuscripts must include a data availability statement. This statement should provide the following information, where applicable:

- Accession codes, unique identifiers, or web links for publicly available datasets
- A description of any restrictions on data availability
- For clinical datasets or third party data, please ensure that the statement adheres to our policy

Deidentified data and study protocols used in this publication will be made available to qualified researchers who provide a valid research question within the scope of the informed consent, and may be subject to a data use agreement. Requests will be responded to within 30 days. Please direct inquiries to the corresponding author (Jean-Cosme Dodart, jc@vaxxinity.com).

Research involving human participants, their data, or biological material

Policy information about studies with human participants or human data. See also policy information about sex, gender (identity/presentation), and sexual orientation and race, ethnicity and racism.

Reporting on sex and gender	Sex/gender (male, female) of participants was determined based on self-report and collected on case report form. Of the 20 enrolled participants, 20% of participants were female and 80% participants were male.
Reporting on race, ethnicity, or other socially relevant groupings	Race (Ethnic or racial group to which subject belongs) include White, Black or African American, Asian, American Indian or Alaska Native, Native Hawaiian or other Pacific Islander, Mixed and Other, were all determined based on self-report and collected on the case report form. All participants were White.
Population characteristics	Criteria for enrollment: Male and female participants with a diagnosis of Parkinson's Disease (PD, as confirmed by a treating general practitioner or neurologist) aged 40 to 85 years, with a body mass index of 18 to 32 kg/m2, who were postmenopausal, surgically sterile or using adequate contraception, with no clinically significant abnormalities. A Dopamine Transporter (DaT) scan was performed if no historic DaT scan was available to confirm the loss of dopaminergic activity as part of the PD diagnosis. Participants were allowed to use concomitant medication for PD or other comorbidities if the regimen was stable before first injection. Further details on the protocol, eligibility criteria, and study design are available on request.
Recruitment	Recruitment was done by identifying participants using an existing database and by using advertisements. Recruitment to this single-center study may have been limited by geographical location.
Ethics oversight	The study was done in accordance with the Declaration of Helsinki and International Council for Harmonisation Good Clinical Practice guidelines. Independent ethics approval for the protocol was granted by the Beoordeling Ethiek Biomedisch Onderzoek (BEBO), Assen, the Netherlands, and all participants provided written informed consent before entering the trial.

Note that full information on the approval of the study protocol must also be provided in the manuscript.

Field-specific reporting

Please select the one below that is the best fit for your research. If you are not sure, read the appropriate sections before making your selection.

☒ Life sciences ☐ Behavioural & social sciences ☐ Ecological, evolutionary & environmental sciences

For a reference copy of the document with all sections, see nature.com/documents/nr-reporting-summary-flat.pdf

Life sciences study design

All studies must disclose on these points even when the disclosure is negative.

Sample size	The sample size was considered adequate to characterize the safety, tolerability, and dose-response profile of UB-312's immunogenicity, based on data from phase 1 in healthy volunteers. The trial was not powered for statistical comparisons between regimens, and results presented for safety and immunogenicity analyses are descriptive
Data exclusions	Safety and tolerability were analyzed based on the safety population defined as all participants randomized and exposed to at least one vaccination, identical to the modified intent-to-treat (mITT) population. The analyzes of immunogenicity and pharmacodynamic endpoints were performed by treatment allocation and based on the per-protocol (PP) population. The PP population was defined as all participants who received all planned vaccinations (up to the point that a protocol violation would take place, if applicable), fulfilled all entry criteria, and had no critical or major protocol deviations that required exclusion of the participant. There were no critical or major protocol deviations. Twenty participants were administered the first and second injection, and 19 participants were administered the third injection; one participant in the 300/100/100 µg cohort was not administered the third vaccination due to a SAE, thereafter, the PP population was 19 of 20 participants.
Replication	This study was not replicated, but CSF samples were analyzed in triplicates in the seed amplification assay and in duplicates in the pS129-aSyn assay. Results from the seed amplification assay demonstrated a high rate of successful replication across triplicate analyses.
Randomization	Eligible participants with PD were randomized by a code generated by SAS version 9·4 to one of two UB-312 treatment cohorts or placebo by an independent statistician, without any restrictions or stratifications. Participants were randomized in a consecutive order starting with the lowest number and were numbered according to the treatment cohort. Both cohorts consisted of ten participants and were randomized seven : three (UB-312: placebo). The planned regimen in cohort 1 was 300/100/100 µg and in cohort 2 300/300/300 µg.
Blinding	Individual randomization codes per participant were placed in a single sealed envelope, labelled 'emergency decoding envelopes' and were kept in a safe cabinet at the clinical site. The randomization code was unblinded/broken and made available for data analysis only after study closure, i.e., when the study has been completed, the protocol deviations determined, and the clinical database declared complete, accurate and locked. Syringes either with either UB-312 or placebo were prepared by an independent, unblinded pharmacist at the Leiden University Medical Centre. Both had an identical white, opaque appearance. Both the participants and the clinical staff at the site were blinded to the treatment during the clinical conduct of the study.

Reporting for specific materials, systems and methods

We require information from authors about some types of materials, experimental systems and methods used in many studies. Here, indicate whether each material, system or method listed is relevant to your study. If you are not sure if a list item applies to your research, read the appropriate section before selecting a response.

Materials & experimental systems

n/a	Involved in the study
☐	☒ Antibodies
☒	☐ Eukaryotic cell lines
☒	☐ Palaeontology and archaeology
☒	☐ Animals and other organisms
☐	☒ Clinical data
☒	☐ Dual use research of concern
☒	☐ Plants

Methods

n/a	Involved in the study
☒	☐ ChIP-seq
☒	☐ Flow cytometry
☒	☐ MRI-based neuroimaging

Antibodies

Antibodies used

A commercial anti-αSyn antibody (BD Bioscience, Cat. No. 610787) was used as positive control for dot blot analyses.
Dilution:
Seed amplification assay:
To determine the optimal dilution across samples and for the evaluation of the kinetic parameters after UB-312 immunization, SAA was performed as described in Concha-Marambio et al., with modifications. The assay included 40 µL of sample and 60 µL of reaction mixture for a final 100 µL reaction comprising 0.3 mg/mL of recombinant αSyn (Amprion, Cat. No. S2020), 500 mM NaCl, 100 mM PIPES pH 6.5, 0.1% sarkosyl, and 2 1/8" silicone nitride beads (Tsubaki Nakashima). To assess the optimal dilution, CSF samples were 3-fold serially diluted in synthetic CSF (Amprion, Cat. No. S2022) up to 1:729 and evaluated in the assay. For the assessment of αSyn-SAA kinetics, CSF samples underwent a single 5-fold dilution in synthetic CSF and were tested in triplicate. For the assessment of αSyn-SAA kinetics, CSF samples underwent a single 5-fold dilution in synthetic CSF and were tested in triplicate.

Measurements of pS129-αSyn in CSF samples:
The concentrations of CSF pS129-αSyn were measured using the Phospho-a-Synuclein S129 kit from MagQu (MagQu, Taiwan, Cat. No. MF-PS1-0060) and Immunomagnetic Reduction (IMR). Before measurement, CSF samples were thawed on ice and reagents were brought to room temperature. CSF was first diluted 20 times with PBS. Thereafter, 60 mL of diluted CSF sample were added to 60 mL of IMR reagent for IMR analysis.

Validation

We used only one antibody and the references listed here support the validation of this antibody.
Jo E, Mclaurin J, Yip CM, St George-Hyslop P, Fraser PE. alpha-Synuclein membrane interactions and lipid specificity. J Biol Chem. 2000; 275(44) :34328-34334. (Clone-specific)
Liu Y, Fallon L, Lashuel HA, Liu Z, Lansbury PT Jr. The UCH-Ll gene encodes two opposing enzymatic activities that affect alpha-synuclein degradation and Parkinson's disease susceptibility. Cell. 2002; 111(2):209-218. (Clone-specific: lmmunoprecipitation, Western blot)
Maroteaux L, Campanelli JT, Scheller RH. Synuclein: a neuron-specific protein localized to the nucleus and presynaptic nerve terminal. J Neurosci. 1988; 8(8):2804-2815.(Biology)
Ostrerova-Golts N, Petrucelli L, Hardy J, Lee JM, Farer M, Wolozin B. The A53T alpha-synuclein mutation increases iron-dependent aggregation and toxicity. J Neurosci. 2000; 20(16):6048-6054. (Clone-specific: lmmunofluorescence, Western blot)
van der Putten H, Wiederhold KH, Probst A, et al. Neuropathology in mice expressing human alpha-synuclein. J Neurosci. 2000; 2 (16):6021-6029. (Clone-specific: lmmunohistochemistry)

Clinical data

Policy information about clinical studies
All manuscripts should comply with the ICMJE guidelines for publication of clinical research and a completed CONSORT checklist must be included with all submissions.

Clinical trial registration

ClinicalTrials.gov: NCT04075318

Study protocol

This clinical trial was conducted at the Centre for Human Drug Research (CHDR), the Netherlands.
Recruitment occurred October 27, 2021, to April 06, 2022. Between January 11, 2022, and April 27, 2022, 21 participants were randomized to either UB-312 or placebo, and one was planned as reserve participant. Promasys database was used to collect and store data. Data transfer agreements were set up for third party data transfers.

Data collection

Trial was conducted at the Centre for Human Drug Research (CHOR), the Netherlands. Promasys database was used to collect and store data. Data transfer agreements were setup for third party data transfers.

Outcomes

The primary endpoints were to evaluate the safety and tolerability as determined by the assessment of TEAEs, safety blood and urine tests, neurological and physical examinations, ECG, and immunogenicity as determined by anti-aSyn antibodies in blood and CSF. The

exploratory objectives were to determine the immunogenicity of UB-312 against components of the vaccine, differences in total aSyn and free aSyn in blood and CSF, effects on MDS-UPDRS and MoCA, and target engagement by detecting misfolded aSyn in CSF using a aSyn seed amplification assay.

Plants

Seed stocks

Report on the source of all seed stocks or other plant material used. If applicable, state the seed stock centre and catalogue number. If plant specimens were collected from the field, describe the collection location, date and sampling procedures.

Novel plant genotypes

Describe the methods by which all novel plant genotypes were produced. This includes those generated by transgenic approaches, gene editing, chemical/radiation-based mutagenesis and hybridization. For transgenic lines, describe the transformation method, the number of independent lines analyzed and the generation upon which experiments were performed. For gene-edited lines, describe the editor used, the endogenous sequence targeted for editing, the targeting guide RNA sequence (if applicable) and how the editor was applied.

Authentication

Describe any authentication procedures for each seed stock used or novel genotype generated. Describe any experiments used to assess the effect of a mutation and, where applicable, how potential secondary effects (e.g. second site T-DNA insertions, mosiacism, off-target gene editing) were examined.
